# STARR‐seq and UMI‐STARR‐seq: Assessing Enhancer Activities for Genome‐Wide‐, High‐, and Low‐Complexity Candidate Libraries

**DOI:** 10.1002/cpmb.105

**Published:** 2019-09-09

**Authors:** Christoph Neumayr, Michaela Pagani, Alexander Stark, Cosmas D. Arnold

**Affiliations:** ^1^ Research Institute of Molecular Pathology (IMP) Vienna Biocenter (VBC) Vienna Austria; ^2^ Medical University of Vienna Vienna Biocenter (VBC) Vienna Austria

**Keywords:** *cis*‐regulatory element, enhancer, functional genomics, gene expression, gene regulation, massively parallel reporter assay, MPRA, STARR‐seq, transcriptional regulation, UMI‐STARR‐seq

## Abstract

The identification of transcriptional enhancers and the quantitative assessment of enhancer activities is essential to understanding how regulatory information for gene expression is encoded in animal and human genomes. Further, it is key to understanding how sequence variants affect enhancer function. STARR‐seq enables the direct and quantitative assessment of enhancer activity for millions of candidate sequences of arbitrary length and origin in parallel, allowing the screening of entire genomes and the establishment of genome‐wide enhancer activity maps.

In STARR‐seq, the candidate sequences are cloned downstream of the core promoter into a reporter gene's transcription unit (i.e., the 3′ UTR). Candidates that function as active enhancers lead to the transcription of reporter mRNAs that harbor the candidates’ sequences. This direct coupling of enhancer sequence and enhancer activity in *cis* enables the straightforward and efficient cloning of complex candidate libraries and the assessment of enhancer activities of millions of candidates in parallel by quantifying the reporter mRNAs by deep sequencing. This article describes how to create focused and genome‐wide human STARR‐seq libraries and how to perform STARR‐seq screens in mammalian cells, and also describes a novel STARR‐seq variant (UMI‐STARR‐seq) that allows the accurate counting of reporter mRNAs for STARR‐seq libraries of low complexity. © 2019 The Authors.

**Basic Protocol 1**: STARR‐seq plasmid library cloning

**Basic Protocol 2**: Mammalian STARR‐seq screening protocol

**Alternate Protocol**: UMI‐STARR‐seq screening protocol—unique molecular identifier integration

**Support Protocol**: Transfection of human cells using the MaxCyte STX scalable transfection system

## INTRODUCTION

Enhancers are genomic *cis*‐regulatory sequences that control transcription in a highly cell type–specific fashion (Heinz, Romanoski, Benner, & Glass, 2015; Shlyueva, Stampfel, & Stark, 2014; Spitz & Furlong, 2012; Visel, Rubin, & Pennacchio, 2009), enabling differential gene expression during development and homeostasis. Indeed, mutations in enhancers impact gene regulation and can lead to various diseases (Murakawa et al., 2016; Smith & Shilatifard, 2014; Visel, Rubin, et al., 2009). Moreover, genome‐wide association studies (GWAS) locate many disease‐associated single‐nucleotide‐polymorphisms (SNPs) to enhancer regions, and to the non‐coding part of the genome more generally (Corradin & Scacheri, 2014). This has led to an increased interest in identifying enhancers and assessing the impact of mutations on the enhancers’ activities. However, in contrast to protein‐coding genes, cell type–specific enhancers lack a common sequence structure. Therefore, the computational prediction of enhancers and their activities, as well as the impact of sequence variants on enhancer activity, has remained challenging (Kleftogiannis, Kalnis, & Bajic, 2016; Lim, Chung, Chong, & Lee, 2018). Consequently, enhancers are typically predicted based on certain chromatin features, including DNA accessibility (open chromatin), enhancer‐associated post‐translational modifications of histones (H3K4me1 and H3K27ac; Calo & Wysocka, 2013; Heintzman et al., 2009), enhancer RNAs (eRNAs; Andersson et al., 2014; Lam, Li, Rosenfeld, & Glass, 2014), and transcription factor and cofactor binding (Spitz & Furlong, 2012; Visel, Blow, et al., 2009a). Although these features correlate with enhancer regions and activities, they are only imperfect predictors (Catarino & Stark 2018; Shlyueva, Stampfel, et al., 2014), and are typically complemented by methods that directly measure enhancer activities.

To identify active enhancers and quantify their activity, a functional readout for enhancer activity is required (Catarino & Stark, 2018). Because enhancers retain their activity outside of their endogenous sequence context (Catarino & Stark 2018; Shlyueva, Stampfel, et al., 2014), the gold standard to assess enhancer activities has been reporter gene assays (e.g., luciferase assays). These assays directly test the ability of a candidate sequence to drive reporter gene transcription, which is quantified by the abundance of the resulting reporter proteins (e.g., luciferase via chemiluminescence). However, these classical reporter‐gene assays are limited in throughput, as candidates have to be tested one‐by‐one. To overcome this limitation, a variety of massively parallel reporter assays (MPRAs) have been developed over the past years that couple a candidate sequence to unique DNA sequences that serve as *molecular barcodes*. This allows the investigator to directly read out reporter transcript abundance by deep sequencing in experiments that assess many candidates in parallel (Inoue & Ahituv, 2015; Santiago‐Algarra, Dao, Pradel, España, & Spicuglia, 2017).

**Figure 1:**
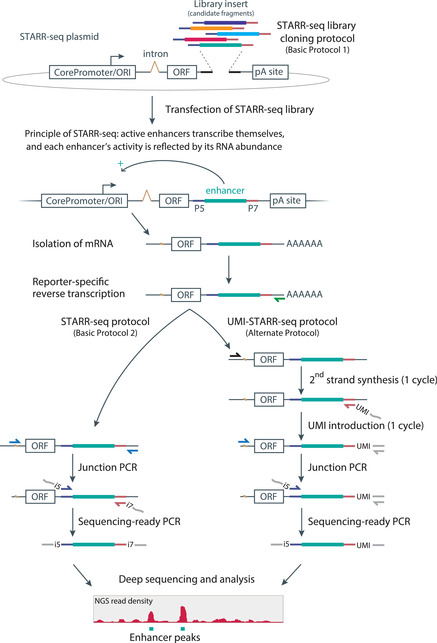
Step‐wise schematic overview of STARR‐seq and UMI‐STARR‐seq. A highly complex library insert (Illumina adapter flanked candidate fragments) is cloned into the 3′UTR of the reporter transcription unit of the STARR‐seq screening plasmid (Basic Protocol 1). After STARR‐seq library transfection into cells, active enhancers transcribe themselves as part of the 3’UTR of the reporter transcripts. The reporter transcripts are isolated as part of the cellular mRNA (isolation of mRNA) and selectively reverse transcribed using a reporter‐specific RT primer (green arrow). Standard STARR‐seq (Basic Protocol 2; left): To selectively amplify reporter cDNAs and not residual input DNA (library plasmid), a nested 2‐step PCR strategy is employed. During the junction PCR step, reporter cDNAs are specifically amplified using a forward primer (junction primer) that exclusively binds to the spliced reporter cDNA. Using the Illumina index primers (i5 and i7), the candidate sequences (Illumina adapter flanked) are amplified during the sequencing‐ready PCR step and subjected to deep sequencing. UMI‐STARR‐seq (Alternate Protocol; right): For low‐complexity candidate libraries, unique molecular identifiers (UMI) can be introduced prior to amplification, allowing the precise counting of reporter mRNAs (Alternate Protocol). To render the reporter cDNAs competent for UMI introduction, second‐strand synthesis is performed with a reporter‐specific forward primer, resulting in double‐stranded reporter DNA. The UMI is introduced by linear PCR with a modified Illumina i7 primer that harbors the UMI at the position of the i7 index. Up to this step, no amplification occurred, such that the UMIs identify individual reporter mRNAs. Only now, the UMI bearing reporter DNAs are amplified, first by the junction PCR and then by the sequencing‐ready PCR. Note that in UMI‐STARR‐seq, indexing is only possible using the i5 index, as the i7 index is replaced by the UMI. Consequently, the UMI is read as Index 1 during deep sequencing on an Illumina platform. STARR‐seq signal over input directly and quantitatively reports on the enhancer activity. ORI, bacterial origin of replication; ORF, open reading frame included in the reporter transcript with no specific function except RNA stabilization. pA site, poly‐adenylation site; P5 and P7, P5 (forward) and P7 (reverse) Illumina adapters.

To identify enhancers and quantitatively measure their strength on a genome‐wide scale, we developed STARR‐seq in *Drosophila melanogaster* cells and initially demonstrated its applicability to focused libraries in human cells (Arnold et al., 2013). In STARR‐seq, a comprehensive library with candidate DNA fragments of arbitrary origin and length is cloned into the 3′UTR of a reporter gene, rendering the candidate sequence part of the reporter‐gene transcription unit. Consequently, if a candidate exhibits enhancer activity, it will activate reporter gene transcription, thereby also transcribing itself as part of the reporter transcript. The abundance of the resulting reporter mRNAs directly reports on each candidate's enhancer activity, and is quantified by deep sequencing. In STARR‐seq, each candidate therefore serves as its own barcode, and this direct coupling of candidate sequence and activity allows the parallel screening of highly complex candidate libraries (Arnold et al., 2013; Fig. 1, left).

The original protocol for STARR‐seq has been successfully used to map cell type–specific (Arnold et al., 2013; Yáñez‐Cuna et al., 2014) or inducible enhancers (Franz, Shlyueva, Brunner, Stark, & Basler, 2017; Shlyueva, Stelzer, et al., 2014) in *Drosophila melanogaster* cell lines. Furthermore, it has led to the identification of enhancer‐core promoter specificity as the basis of two fundamentally different transcriptional regulatory programs (Zabidi et al., 2015). It has also been successfully used by our laboratory and others for the parallel assessment of medium‐size and large candidate pools in human cell lines (Johnson et al., 2018; Vanhille et al., 2015; Wang et al., 2018), revealing insights into stem cell enhancers (Barakat et al., 2018), acquisition of resistance to treatment in cancer cells due to transcriptional network shifts (Rathert et al., 2015), active p53‐target enhancers (Verfaillie et al., 2016), and glucocorticoid receptor dependent enhancers (Vockley et al., 2016).

We recently adapted the STARR‐seq protocol for genome‐wide screening in human cell lines (Muerdter et al., 2018), which required a solution to two problems that apply to all episomal reporter assays in mammalian cells involving plasmid transfection: the activation of a type I interferon response upon plasmid transfection (Chen, Sun, & Chen, 2016; Muerdter et al., 2018; Nejepinska, Malik, Wagner, & Svoboda, 2014) and the aberrant transcription initiation from the bacterial origin of replication (ORI) on reporter plasmids (Lemp, Hiraoka, Kasahara, & Logg, 2012; Muerdter et al., 2018). The new/adapted protocol allows genome‐wide enhancer‐activity screens in human cells and has enabled the identification of genome‐wide sets of constitutively active and interferon‐inducible enhancers in HeLa cells (Muerdter et al., 2018).

Here, we provide a step‐by‐step procedure to perform STARR‐seq in human cell lines, including the cloning of medium‐size (or “focused”) libraries derived from bacterial artificial chromosomes (BACs) and highly complex genome‐wide libraries (Basic Protocol 1), as well as transfection of human cells, RNA processing, and reporter transcript amplification for deep sequencing (Basic Protocol 2; Fig. 1, left). Additionally, we provide advice on critical steps and present a novel protocol variant, UMI‐STARR‐seq (Alternate Protocol), which uses unique molecular identifiers (UMIs) to enable the quantification of reporter transcripts for candidate libraries of low complexity (e.g., pools of individual candidates or synthesized DNA oligo pools; Fig. 1, right).

## STRATEGIC PLANNING

### Considerations when Establishing STARR‐seq

To establish STARR‐seq, we recommend using a focused candidate library from BAC DNA. The selected BACs need to include known enhancers that are active in the cell line of interest to serve as positive controls. Their detection by STARR‐seq indicates successful performance. Due to their lower complexity, focused libraries also make it possible to perform STARR‐seq on a much smaller scale (at least 10‐fold), using much fewer cells compared to genome‐wide libraries. This facilitates the establishment and optimization of the STARR‐seq protocol for the cell line of interest. We advise investigators to always perform a focused STARR‐seq screen prior to scaling up to genome‐wide STARR‐seq screens.

### Choice of STARR‐seq Screening Plasmid

For human STARR‐seq, we recommend using the mammalian STARR‐seq plasmid (hSTARR‐seq_ORI vector; Addgene #99296) from Muerdter et al. (2018). This plasmid uses the bacterial ORI as core promoter [see Background Information and Muerdter et al. (2018) for details]. Compared to the first‐generation human STARR‐seq plasmid (SCP1 as core promoter; pSTARR‐seq_human; Addgene #71509), this plasmid provides the strongly improved signal‐to‐noise levels required for genome‐wide screens.

For fly STARR‐seq, we recommend using the Drosophila STARR‐seq plasmids from Arnold et al. (2013) and Zabidi et al. (2015), depending on which enhancer type will be assessed (developmental versus housekeeping: pSTARR‐seq_fly (Addgene #71499) or pSTARR‐seq_fly‐RpS12 (Addgene #71504), respectively).

*IMPORTANT NOTE*: The human and the fly STARR‐seq plasmids contain different introns and therefore require different primers for the junction PCR (Basic Protocol 2, steps 127 to 130). These primers span the exon‐exon‐splice junction and do not align to the STARR‐seq plasmid sequence.

### STARR‐seq Plasmid Library

The candidate fragments in STARR‐seq libraries can be of arbitrary length, limited only by cloning efficiency. We frequently use lengths from 150 to 1500 bp and recommend 1200 to 1500 bp for human STARR‐seq. To avoid library distortion during the PCR amplification steps and deep sequencing, we recommend keeping the length range of the candidates within an individual STARR‐seq library rather narrow (within approximately ∼300 bp). The candidate sequences can be obtained from arbitrary sources of DNA (Arnold et al., 2013), including BAC and genomic DNA, DNA fragments enriched for regions of interest (e.g., via fragment capture, ChIP or similar), and synthesized DNA oligo pools. When performing STARR‐seq with libraries of very low complexity (e.g., individual candidates, synthesized oligo pools, or pools of pre‐selected or pre‐enriched candidates), we recommend using the UMI‐STARR‐seq screening protocol (Alternate Protocol), as low‐complexity libraries are more susceptible to PCR amplification biases and other distortions. To prevent such biases and distortions, the UMI‐STARR‐seq protocol employs a unique‐molecular‐identifier (UMI), which allows the counting of individual reporter transcript molecules, enabling their precise quantification.

### Choice of Cell Line and Transfection Method

If possible, use a cell line that is highly transfectable and easy to culture (both adherent and suspension cell lines are suitable for STARR‐seq). The transfection efficiency may vary depending on cell line, transfection method, and protocol. Before performing STARR‐seq, establish a transfection protocol for the cell line of interest, which yields high transfection efficiency (recommended >60%) and cell viability. Determine the transfection efficiency by transfecting a control plasmid that expresses GFP (or any other fluorescent protein) under the control of a strong promoter (e.g., CMV) and quantifying the fraction of GFP‐positive cells by FACS analysis—higher transfection efficiency usually results in stronger STARR‐seq signals. We recommend electroporation to achieve high transfection efficiencies, and regularly perform STARR‐seq using plasmid library electroporation in HeLa S3 (use INF inhibitors; see below) and HCT116 cells. Chemical transfection (Barakat et al., 2018; Vockley et al., 2016) as well as lentiviral or adeno‐associated virus (AAV) transfection (infection) should also be applicable (Inoue et al., 2017; Maricque, Dougherty, & Cohen, 2017; Nguyen et al., 2016; Shen et al., 2016) with appropriately adjusted STARR‐seq plasmids.

Note that low transfection efficiency or cell viability may limit the performance of STARR‐seq. For cell lines that are difficult to transfect by any method, an increase in cell number used for STARR‐seq could compensate for the low transfection efficiency.

### Interferon Response in Mammalian Cells Post Plasmid Transfection

The transfection of plasmid DNA triggers a type I interferon (INF‐I) response in many mammalian and human cell lines via the cGAS/STING pathway (Muerdter et al., 2018; Paludan & Bowie, 2013), making it necessary to evaluate the cell line of interest prior to performing STARR‐seq [see Muerdter et al. (2018)]. An active interferon response would lead to false‐positive and false‐negative enhancer activities (see Background Information for details). To prevent the mounting of an interferon response, inhibitors for the key kinases of the cGAS/STING pathway (PKR and TBK1; see Materials) should be used.

To get more detailed background information regarding the use of the ORI as core promoter, different cell lines eliciting an type‐I interferon response, or alternative STARR‐seq strategies, see Muerdter et al. (2018).

## Basic Protocol 1 STARR‐seq PLASMID LIBRARY CLONING

Due to the unique location of the candidate fragments within the reporter gene transcription unit of the STARR‐seq screening plasmid, each candidate serves as its own *barcode*, which enables the straightforward and simple cloning of candidate libraries. The transcription unit consists of a core promoter followed by an intron and a reporter gene (ORF, a truncated form of GFP). The candidate fragments are cloned between the ORF and the poly(A) site, i.e., into the 3′UTR of the reporter gene (Fig. 1 top). For human STARR‐seq, we recommend using the second‐generation human STARR‐seq screening plasmid from Muerdter et al. (2018) (hSTARR‐seq_ORI vector; Addgene #99296). Compared to the first‐generation human STARR‐seq screening plasmid (SCP1 as core‐promoter; pSTARR‐seq_human; Addgene #71509), this plasmid exhibits strongly improved signal‐to‐noise levels, as it uses the bacterial ORI as core promoter [see Background Information and Muerdter et al. (2018) for details].

To enable highly efficient library cloning and avoid the cleavage of candidates, we clone the STARR‐seq plasmid library by recombination (Gibson assembly is also possible). This requires that the candidate fragments be flanked by constant sequences that match to the plasmid backbone sequences around the insertion site. To ease cloning and the final deep sequencing on the Illumina platforms, we first ligate the Illumina adapters (here we use the NEBNext hairpin‐adapters) to randomly sheared and size‐selected BAC or genomic DNA or pre‐enriched candidate pools. As the Illumina adapter is a Y‐shaped adapter (double‐stranded on one end), candidates will be ligated in both orientations and will, in the resulting library, be present in both orientations (roughly equimolar). In principle, any source of DNA is applicable (Arnold et al., 2013). When using synthesized DNA oligo pools, we recommend including the adapter sequences into the DNA oligos. This allows the PCR amplification of the DNA oligo pool and the directional cloning of all candidates (note that the adapter sequences have to be included directionally, i.e., the P5 adapter sequence at the 5′ end and the reverse‐complement P7 adapter sequence at the 3′ end).

Also, the length of the candidate fragments can be of a wide range, essentially limited only by cloning efficiencies. We have used fragments between 150 and 1500 bp, and we recommend 1200 to 1500 bp for human STARR‐seq. Note, however, that an individual library should only contain inserts of a limited size range of 100 to 300 bp around the desired fragment length, as the various amplification and deep sequencing steps can distort the length distribution. Note that the source of DNA, i.e., the input material for the library insert, is the major determinant of library complexity.

The adapter‐ligated DNA is then PCR amplified using primers that bind to the Illumina adapters (constant part of the insert) and add homology arms for directional cloning into the STARR‐seq screening plasmid. Importantly, the candidate fragments cloned via Y‐linker adapters will become directional at this PCR step, such that candidates cloned in either direction can be unambiguously identified by deep sequencing. Next, the library insert is cloned by recombination into the STARR‐seq screening plasmid using the homology arms added to the Illumina adapters during library insert amplification. As the cloning efficiency strongly impacts library complexity, it is necessary to ensure the highest possible efficiency.

Third, the library cloning reactions are column purified and transformed into competent bacteria, which are subsequently grown in liquid cultures for the large‐scale amplification of the STARR‐seq plasmid library. A high transformation efficiency is critical for the complexity and quality of the library (especially for genome‐wide libraries). Therefore, transformation should be performed by electroporation using electrocompetent bacteria with the highest efficiency.

This protocol describes how to clone a medium‐sized focused library from BAC‐derived DNA fragments and a highly complex genome‐wide library.

### Materials

Genomic DNA (e.g., Promega, cat. no. G3041; or any other source of genomic DNA; for genome‐wide library)
BAC DNA [e.g., BAC PAC Resource Center (BPRC); for focused library]
LB medium (Elbing & Brent, 2018)
Chloramphenicol
Large‐Construct Kit (Qiagen cat. no. 12462; focused library only)
1× TE buffer (10 mM Tris·Cl, pH 8.0, 1 mM EDTA; Moore, 1996)
6× DNA loading dye (see Current Protocols article: Voytas, 2000)
1‐kb DNA ladder (should indicate 1 and 1.5 kb)
SYBR® Safe DNA Gel Stain (Invitrogen cat. no. S33102)
Gel Extraction Kit (Qiagen cat. no. 28704)
EB (10 mM Tris·Cl, pH 8.5)
NEBNext® Ultra™ II DNA Library Prep Kit for Illumina® (NEB cat. no. E7645S)
NEBNext Multiplex Oligos for Illumina (NEB cat. no. E7335L)
Ethanol
MonoQ H_2_O
AMPure XP beads (Beckman Coulter cat. no. A63882)
RNase‐free H_2_O (Qiagen cat. no. 129115)
QIAquick PCR Purification Kit (Qiagen cat. no. 28104)
Primers:
  library cloning forward (fw) primer:
    TAGAGCATGCACCGGACACTCTTTCCCTACACGACGCTCTTCCGATCT
  library cloning reverse (rev) primer:
    GGCCGAATTCGTCGAGTGACTGGAGTTCAGACGTGTGCTCTTCCGATCT
  Illumina‐compatible i5 and i7 index primers
KAPA 2× HiFi HotStart ReadyMix (Kapa Biosystems cat. no. KK2601)
STARR‐seq screening plasmid (see Strategic Planning, “Choice of STARR‐seq screening plasmid)
*Age*I‐HF restriction enzyme (NEB cat. no. R3552L)
*Sal*I‐HF restriction enzyme (NEB cat. no. R3138L)
10× CutSmart buffer (NEB cat. no. B7204S)
MinElute PCR Purification Kit (Qiagen cat. no. 28004)
In‐Fusion® HD Cloning Kit (Clontech cat. no. 639650); alternatives are:
  Gibson Assembly Master Mix (NEB cat. no. E2611L)
  NEBuilder HiFi DNA Assembly (NEB cat. no. E2621L)
MegaX DH10B™ T1R electrocompetent bacteria (Invitrogen cat. no. C640003)
LB agar plates (Elbing & Brent, 2018)
Ampicillin
Plasmid Plus Giga Kit (Qiagen cat. no. 12991)

37°C heating block
Shaker
UV/Vis spectrophotometer
Refrigerated centrifuge
Covaris S220 focused ultrasonicator
Covaris microTUBEs with AFA fiber (Covaris cat. no. 520045)
Blue light illuminator (Thermo Fisher cat. no. G6600EU)
Scalpel
PCR strips (Biozym cat. no. 711030)
PCR 8er‐CapStrips (Biozym cat. no. 711040)
Magnetic separator for PCR strips (NEB cat. no. S1515S)
Magnetic separator for 1.5‐ml and 2‐ml tubes (Thermo Fisher cat. no. 12321D)
Thermal cycler (PCR machine)
1.5‐ml DNA LoBind tubes (Eppendorf cat. no. 0030108051)
Gene Pulser Xcell Electroporation System (BioRad)
MicroPulser cuvettes, 0.1‐cm gap (Bio Rad cat. no. 1652089)
14‐ml polypropylene round‐bottom tubes
50‐ml conical tubes (e.g., Corning Falcon)
5 L baffled Erlenmeyer flasks
Bacteria shaker with holders for 5‐L Erlenmeyer flasks
1‐L centrifugation bottles

Additional reagents and equipment for quantitation of DNA (see Current Protocols article: Gallagher & Desjardins, 2006) and agarose gel electrophoresis (see Current Protocols article: Voytas, 2000)

### Preparation of BAC and genomic DNA

#### Culturing BAC clones and extraction of BAC DNA

1 Select the genomic loci of interest. Include genomic loci that span known enhancers that are active in the cell line of interest. These enhancers serve as positive controls in STARR‐seq.
  See Strategic Planning for more details.
  If generating a genome‐wide library, skip steps 1 to 11.
2 Identify BAC clones that cover the genomic loci of interest.
  The number of BACs per library is variable. We have successfully cloned and screened libraries from up to 70 BACs. We anticipate that higher numbers of BACs are also applicable.
3 Pre‐culture each BAC clone individually in 5 ml LB medium containing 12.5 µg/ml chloramphenicol for at least 12 hr at 37°C while shaking at 200 rpm.
4 Inoculate each BAC pre‐culture (5 ml) into 50 ml LB medium + chloramphenicol (12.5 µg/ml final concentration).
  The minimum volume of the pooled BAC culture that is required for BAC DNA extraction (step 9) is 500 ml.
  If using less than 10 BAC clones, increase the volume per culture accordingly, to ensure a final volume of 500 ml for the pooled BAC culture.
5 Grow BAC cultures individually for at least 16 hr at 37°C while shaking at 200 rpm.
6 Measure OD_600_ for each BAC culture using an appropriate spectrophotometer.
7 Pool BAC cultures equally according to OD_600_ measurements.
  Determine the required volume per BAC culture according to the OD_600_ measurements. This results in pooling equal amounts of bacteria and an even representation of each BAC within the BAC DNA pool.
8 Divide the BAC culture pool into 500‐ml batches (if applicable) and spin down for 15 min at 4000 × *g*, 4°C, to pellet the bacteria.
  Bacterial pellets can be stored at −20°C.
9 Extract BAC DNA using the Qiagen Large‐Construct Kit according to the manufacturer's protocol.
10 Resuspend BAC DNA in 300 µl 1 × TE buffer.
  Add 300 µl 1 × TE buffer to the BAC DNA pellet and incubate at room temperature for at least 12 hr under constant agitation.
11 Measure the concentration of the BAC DNA pool (Gallagher & Desjardins, 2006).

### Sonication and size‐selection of human BAC and genomic DNA

12 Sonicate DNA to a target size of 1200 to 1500 bp.
  Example settings when using Covaris microTUBEs with AFA fiber and a Covaris S220 focused‐ultrasonicator:
  For a target size of 1200 to 1500 bp, sonicate for 15 s per 5 µg DNA with intensity parameters: 2 “duty cycle”; 4 “intensity”; 200 “cycles/burst.”
  For a target size of 500 to 750 bp, sonicate for 45 s per 5 µg sample with intensity parameters: 5‐3‐200.
13 Use 5 µg DNA (BAC or genomic DNA) in 130 µl TE buffer per sonication.
  Sonicate 10 µg BAC DNA or 50 µg genomic DNA in total (you will recover ∼10% DNA after sonication and size selection).
  Prepare two samples of sonicated DNA for a focused library or ten samples for a genome‐wide library.
14 Collect the sonicated DNA on ice.
15 Pool all sonicated DNA samples and add the corresponding volume of 6× DNA loading dye.
16 Load 12 µl of a 1‐kb DNA ladder in both outermost wells of a 1% agarose gel (well size: 60 µl). See Current Protocols article Voytas (2000) for essential agarose gel electrophoresis protocols.
  Use SYBR® Safe DNA stain.
  It is important to load the DNA ladder on both sides of the gel (see step 19).
17 Load 60 µl of sonicated DNA per well on the 1% agarose gel and perform electrophoresis at 140 V for 20 to 30 min.
  Do not load more than 2.5 µg DNA per well on the gel, to avoid poor separation of the sonicated DNA.
18 Visualize DNA on a blue light illuminator.
  Do not use UV light, as this may lead to DNA damage.
19 Size‐select the sonicated DNA between 1.2 and 1.5 kb.
  Use a ruler or similar implement to connect the respective bands of the DNA ladder on both sides of the gel. Cut the gel along the ruler between 1.2 and 1.5 kb using a scalpel. Cut first at 1.2 kb and then at 1.5 kb.
20 Extract the size‐selected DNA from two gel slices per QIAquick column using the Qiagen Gel Extraction kit according to the manufacturer's protocol.
  You will need 10 columns for genomic DNA and 4 columns for BAC DNA.
  Do not use more than 400 mg agarose per column.
  Dissolve gel slices at 25° to 37°C under constant agitation (300 rpm) for 10 to 15 min. Do not heat to 50°C.
21 Elute the DNA in 50 µl EB.
22 Repeat the elution with the eluate from the first elution (step 21).
  This step increases the yield of purified DNA.
23 Pool the elution fractions from five columns.
24 Purify the gel‐extracted DNA pool from five columns (from step 23) using one QIAquick column according to the manufacturer's protocol for PCR purification.
25 Elute in 50 µl EB and repeat the elution with the eluate.
26 Pool all eluates from step 25.
27 Measure the concentration of the sonicated, size‐selected and purified DNA pool (see Current Protocols article: Gallagher & Desjardins, 2006).

### Generation of focused and genome‐wide library inserts (Illumina adapter ligation)

28 Perform adaptor ligation using the NEBNext Ultra™ II DNA Library Prep Kit for Illumina.
  Use 1 µg sonicated and size‐selected DNA as starting material per reaction.
  Perform one adaptor ligation reaction for a focused library insert and five for a genome‐wide library insert.
29 Precisely follow section 1.1 (NEBNext End Repair) and 1.2 (Adaptor ligation) of the NEBNext Ultra™ II DNA Library Prep Kit for Illumina manual for adaptor ligation.
  After Section 1.2.5 in the manual, continue with step 30 (see below).
  Use NEBNext hairpin adapters and the USER enzyme [both contained in NEBNext Multiplex Oligos for Illumina (E7335L)] and follow the manufacturer's instructions.
  The NEBNext Ultra II Ligation Mixture needs to be cleaned up twice with AMPureXP beads. The first cleanup (steps 30 to 41) recovers all DNA; the second cleanup excludes adapter dimers (steps 42 to 45).

### Cleanup of adaptor‐ligated DNA with AMPureXP beads

Before starting:

Prepare 80% ethanol (diluted with nuclease‐free MonoQ H_2_O)
Warm AMPureXP beads to room temperature.

30 Add 90 µl of the NEBNext Ultra II Ligation Mixture from step 29 per well of a PCR strip.
  The NEBNext Ultra II Ligation Mixture is very viscous.
  Prepare one reaction for a focused library and five reactions for genome‐wide libraries.
31 Add 1.8 vol AMPureXP beads (162 µl) to 1 vol DNA (90 µl) in a well of the PCR strip, vortex, and pipette up and down 20 times.
  Resuspend beads thoroughly by vortexing before use.
32 Incubate for 10 min at room temperature.
33 Transfer the PCR strip to a magnetic separator and incubate for 5 min at room temperature.
  Allow the beads to migrate to the magnet‐touching wall of the tube. Ensure that the solution is clear before removing the supernatant.
  Keep samples on magnetic separator for the following steps (until step 38),
34 Remove the supernatant completely.
  Keep supernatant until the successful cleanup is confirmed.
35 Wash the beads twice with 250 µl of 80% ethanol, incubating in the ethanol for 2 min between the washes.
  Make sure that the beads are completely covered with 80% ethanol.
  Remember to keep samples on the magnetic separator.
36 After the second wash, completely remove the ethanol.
37 Dry the beads at room temperature for 2 to 5 min to allow the evaporation of the remaining ethanol.
  Keep the tubes on the magnet with open lids while drying.
  Avoid over‐drying and elute as soon as the bead pellet starts to show cracks.
38 To elute, add 100 µl RNase‐free H_2_O, close the PCR strip, and remove it from the magnet.
39 Resuspend the beads completely by thoroughly vortexing and incubate at 37°C on a heating block for 3 min while shaking (300 rpm).
  Alternatively, a thermal cycler can be used.
40 Transfer the PCR strips immediately onto the magnetic separator and carefully tilt the magnet to allow the beads to migrate up the tube wall. Incubate for 1 min.
41 Transfer the adapter‐ligated DNA to a new PCR strip.

### Removal of adapter dimers using AMPure XP beads

42 Size‐select the adapter‐ligated DNA from step 41 using AMPure XP beads.
43 Repeat steps 30 to 41 but add 0.8 vol beads to 1 vol DNA (use 80 µl beads for 100 µl DNA).
  Using 0.8 vol of beads per 1 vol DNA selectively excludes DNA fragments <100 to 200 bp, thereby removing adapter dimers. The ratio of beads to DNA sample should be determined for every new batch of AMPure XP beads before first use for size‐selection.
44 Elute the adapter‐ligated DNA in 20 µl EB.
45 Pool all adapter‐ligated DNA samples prior to PCR amplification.
  This applies to genome‐wide libraries only (five samples).

### Test PCR amplification of the adapter‐ligated DNA library insert

46 Perform two test PCR reactions, with 5 and 9 cycles.
  The test PCR is performed to determine the optimal number of PCR cycles that are needed for library insert amplification based on band intensity (typically 6 to 10 cycles) on a 1% agarose gel (see Basic Protocol 2, step 149). It is important to avoid over‐amplification, as this leads to poor‐quality library inserts. Over‐amplification results in the concatemerization of PCR products, which can be monitored by the increase in size of the PCR product after gel electrophoresis.
47 Prepare the test PCR reaction mix (for one reaction):
  1 µl adapter‐ligated DNA (from step 45)
  2.5 µl library_cloning_fw (10 µM)
  2.5 µl library_cloning_rv (10 µM)
  25 µl KAPA 2× HiFi HotStart Ready Mix
  19 µl H_2_O.
  IMPORTANT NOTE: It is critical to use the KAPA HiFi DNA polymerase (KAPA 2× HiFi HotStart Ready Mix) for library insert amplification, to prevent amplification biases. The library_cloning primers (see Materials, above) add homology arms to the library insert, which are needed for cloning the library insert into the STARR‐seq screening plasmid by In‐FusionHD or Gibson cloning.
48 Run the test PCR using the following PCR program:

**Table:** 

1 cycle:	45 s	98°C	(initial denaturation)
5 or 9 cycles:	15 s	98°C	(denaturation)
	30 s	65°C	(annealing)
	45 s	72°C	(extension)
1 cycle:	120 s	72°C	(final extension).
  Extension time depends on insert size, here 1200 to 1500 bp.
49 Analyze the test PCR by gel electrophoresis (Voytas, 2000).
  Run 10 µl PCR plus 2 µl 6× DNA loading dye per test PCR on an 1% agarose gel at 140 V for 15‐30 min. The number of PCR cycles is determined by band intensity. Please see Basic Protocol 2, step 149, and Fig. 2.

### PCR amplification of the adapter‐ligated DNA library insert

50 Set up PCR reactions to amplify the library insert using 1 µl of adapter‐ligated DNA per 50 µl PCR reaction (refer to step 47 for the reaction mixture).
  Perform 30 PCR reactions for the amplification of adapter‐ligated genomic DNA.
  For focused libraries, perform four PCR reactions.
51 PCR amplify the library insert using the number of cycles determined during test PCR.
  See step 48 for the PCR program.
  Scale the PCR reaction mix according to library complexity (i.e. focused versus genome‐wide).

### Purification and size‐selection of the amplified library insert with AMPureXP beads

52 Pool four PCR reactions in a 1.5‐ml DNA LoBind tube for a focused library or pool ten PCR reactions per tube for a genome‐wide library.
  Clean up 3 × 10 PCR reactions for genome‐wide libraries.
  Do not clean up more than 10 PCR reaction in one tube.
53 Purify PCR reaction with AMPure XP beads (see steps 30 to 41, above), using 0.8 vol beads per 1 vol PCR reaction.
  This ratio of beads to PCR selectively excludes DNA fragments <100 to 200 bp.
54 Elute in 10 µl EB per PCR reaction.
  Total volume: 40 µl or 3 × 100 µl for focused or genome‐wide library inserts, respectively.

### PCR purification of the library insert with the QIAquick PCR purification kit

55 Clean up 40 or 100 µl library insert per QIAquick column (according to manufacturer's instructions).
  This step increases the cloning efficiency of the In‐FusionHD reaction.
56 Elute in 50 µl EB.
57 Re‐apply the eluate to the column and elute again.
58 Measure the DNA concentration (Gallagher & Desjardins, 2006).
  For genome‐wide library inserts, pool the elutions from all three columns.

### Restriction digest and purification of the STARR‐seq screening plasmid

59 Prepare restriction digest master mix (genome‐wide library):
  25 µg STARR‐seq screening plasmid (5 µg plasmid for focused library)
  25 µl *Age*I‐HF
  25 µl *Sal*I‐HF
  50 µl CutSmart buffer (10×)
  Make up with H_2_O to 500 µl
  Mix thoroughly by vortexing and distribute 10 × 50 µl master mix to PCR strips.
60 Incubate for 2 hr at 37°C in thermal cycler.
61 Heat inactivate for 20 min at 65°C in thermal cycler.

### Gel purification of the digested STARR‐seq screening plasmid

62 Add 10 µl of 6× DNA loading dye to 50 µl restriction digest reaction.
63 Run 60 µl sample per well on a 1% agarose gel at 160 V for 30 min (Voytas, 2000).
  Run 10 µl 1 kb DNA ladder on the gel.
  Use SYBR® Safe DNA stain.
64 Size‐select the digested plasmid DNA (∼3 kb).
  Visualize the plasmid DNA using blue light.
  Confirm that the restriction digest was complete.
  Cut out the plasmid DNA band (∼3 kb) on a blue light illuminator.
65 Extract the size‐selected plasmid DNA from two gel slices per QIAquick column according to manufacturer's instructions.
  Do not use more than 400 mg agarose per column.
66 Wash twice with 750 µl buffer PE (from QIAquick Kit).
67 Elute twice using 50 and then 25 µl EB.

### PCR purification of the digested STARR‐seq screening plasmid

68 Pool all eluates of the gel extracted plasmid (step 67) and equally divide into two samples.
69 Clean up each sample with one QIAquick column (according to manufacturer's instructions).
  Increased purity of the plasmid DNA used for In‐FusionHD cloning increases the cloning efficiency.
70 Elute twice using 50 and then 25 µl EB.

### MinElute PCR purification of the digested STARR‐seq screening plasmid

71 Pool all eluates from the PCR purification step (step 70) and equally divide into two samples.
72 Clean up with two MinElute columns according to manufacturer's instructions.
  This cleanup step further increases purity and concentrates the digested plasmid DNA.
73 Elute twice, each time using 15 µl EB.

### Library cloning reaction using In‐Fusion HD

*NOTE*: Gibson Assembly or NEBuilder HiFi DNA Assembly can also be used for the library cloning reaction (according to manufacturer's instructions), using the same amounts of plasmid DNA and library insert as indicated for In‐Fusion HD initially (step 74). Cloning efficiency needs to be determined experimentally before library cloning. When experiencing a low cloning efficiency, first switch to alternative cloning methods

74 Prepare In‐Fusion HD master mix (for one reaction):
  125 ng *Age*I‐HF/*Sal*I‐HF‐digested STARR‐seq screening plasmid (from step 73)
  2× molar excess purified library insert (step 58)
  2 µl 5× In‐Fusion HD Enzyme Premix
  Make up with H_2_O to 10 µl
  Use a 2:1 molar ratio of library insert [∼1250 bp (including adapters)] to digested STARR‐seq screening plasmid (∼3 kb). Especially for Gibson Assembly, other molar ratios might also work.
  Perform 40 In‐Fusion HD reactions for a genome‐wide library (four reactions can be processed as pool).
  You can also first perform 20 reactions, clone the library, and determine its complexity. If the library is not complex enough to properly cover the entire genome, perform another 20 reactions. You can pool both libraries.
  We recommend to only use 20 reactions at a time for bacterial transformation, i.e., perform the transformation twice per genome‐wide library.
  Perform four reactions for a focused library (process as pool).
75 Distribute the In‐Fusion HD master mix to PCR strips (40 µl per well, i.e., four pooled reactions).
76 Incubate for 15 min at 50°C in thermal cycler.
77 Place samples on ice.

### MinElute PCR purification of the library cloning reaction

78 Make up In‐Fusion HD reaction pool (four reactions) to 100 µl.
  Add 60 µl H_2_O to 40 µl In‐Fusion HD reaction pool.
79 Clean up one In‐Fusion HD reaction pool per one Qiagen MinElute column according to manufacturer's instructions.
  The purification of the In‐Fusion HD cloning reaction is absolutely essential for high transformation efficiency, and therefore library complexity.
80 Wash twice with 750 µl buffer PE (from Qiagen MinElute Kit).
81 After washing, centrifuge columns at maximum speed for 5 min to dry the membrane.
82 Elute in 12.5 µl EB.
83 Re‐elute by passing the first eluate (12.5 µl) through the column again.
84 Pool all purified library cloning reactions
  This only applies to a genome‐wide library (5 × 12.5 µl).

### Transformation of electrocompetent MegaX DH10B bacteria (Invitrogen)

*NOTE*: Transform bacteria in the afternoon, grow them overnight, and harvest the next morning. Avoid growing them for more than 14 to 16 hr.

Grow 12 to 24 L for a genome‐wide library and 4 L for a focused library. The scale of the liquid culture depends on the demand of the library. We did not experience any influence of the scale of the liquid culture on transformation efficiency.

Before starting:

Pre‐warm LB medium at 37°C (1 day in advance).
Pre‐warm recovery medium (comes with the bacteria) at 37°C.
Pre‐cool 20 µl tips at 4°C.
Pre‐cool DNA1.5 ml LoBind tubes on ice.
Pre‐cool MicroPulser Cuvettes (0.1‐cm gap) on ice.
For maximum efficiency, it is essential to use MegaX DH10B™ T1R electrocompetent bacteria (Invitrogen cat. no. C640003) and precisely follow the protocol (steps 85 to 108).

*NOTE*: Perform all steps on ice and work in cold room (if possible).

85 Distribute 2.5 µl of purified and pooled library cloning reaction (for 20 transformations) to pre‐cooled 1.5‐ml LoBind tubes.
  Perform 20 transformations for a genome‐wide library at once. Perform 40 in total.
  Perform four transformations for a focused library.
86 Pipet 1 µl transformation control plasmid (pUC19) into a pre‐cooled 1.5‐ml LoBind tube.
  pUC19 comes with the bacteria.
87 Thaw five tubes (one tube) of MegaX DH10B™ T1R electrocompetent bacteria on ice.
  It is absolute essential to thaw the bacteria on ice and keep them on ice after thawing.
88 Add 20 µl MegaX DH10B bacteria into each 2.5‐µl library cloning reaction tube and mix carefully by flicking two to three times.
  IMPORTANT NOTE: NEVER pipet MegaX DH10B bacteria up and down.
89 Transfer bacteria‐DNA mix into pre‐cooled MicroPulser cuvettes using pre‐cooled 20 µl tips.
  Make sure to transfer the bacteria without generating bubbles. Distribute the bacteria at the bottom of the cuvette by flicking or tapping.
90 Electroporate bacteria using the Bio‐Rad Gene Pulser Xcell electroporator.
  SETTINGS: 2 kV, 25 µF, 200 Ω, 1 mm.
91 **Immediately** add 1 ml of pre‐warmed (37°C) recovery medium to the cuvette and transfer resuspended bacteria to a 14‐ml polypropylene round‐bottom tube.
  The recovery medium comes with the MegaX DH10B™ T1R electrocompetent bacteria.
  It is critical to immediately add the pre‐warmed medium. Efficiency drops on the order of seconds.
92 Recover the transformed bacteria in 14‐ml polypropylene round‐bottom tubes for 1 hr at 37°C while shaking (>300 rpm).
  Recovering the transformed bacteria in 14‐ml round bottom tubes increases efficiency, compared to recovery in 1.5 ml tubes.
93 Pool all transformation reactions (∼22 to 23 ml) in a 50‐ml conical tube.
94 Prepare a dilution series of the pooled bacterial culture and the transformation control (pUC19). Determine the transformation efficiency of the MegaX DH10B bacteria using the colony counts of pUC19 (also see manufacturer's instructions). Efficiency should be >3 × 10^10^.
  To estimate the library complexity, use colony counts from library transformed bacteria:
  1:10, 100 µl bacteria culture + 900 µl LB medium without addition of antibiotics
  1:50, 200 µl from 1:10 dilution + 800 µl LB medium without addition of antibiotics
  1:500, 100 µl from 1:50 dilution + 900 µl LB medium without addition of antibiotics
  1:5000, 100 µl from 1:500 dilution + 900 µl LB medium without addition of antibiotics.
95 Plate 100 µl of 1:50, 1:500, and 1:5000 dilutions on selective LB agar plates containing 100 µg/ml ampicillin (see Elbing & Brent, 2018).
  More than 50 colonies should be obtained from the 1:5000 dilution of the library transformed bacteria.
96 Add equal volumes of the pooled bacteria culture from step 93 to 2 L pre‐warmed (37°C) LB medium containing 100 µg/ml ampicillin in 5‐L Erlenmeyer flasks.
97 Incubate overnight (∼13 hr) while shaking at 200 rpm at 37°C. Measure OD_600_ after ∼10 to 12 hr.
  OD_600_ should be between 2 and 2.6 when harvesting the bacteria culture.
98 Spin down bacteria culture in 1‐L centrifugation bottles for 30 to 45 min at >4200 × *g*, 4°C.
99 Decant the supernatant, leaving behind ∼10 ml medium for resuspension.
100 Resuspend the bacteria pellets in the residual medium by vortexing and pipetting in the centrifugation bottles.
101 Pool all resuspended bacteria in one centrifugation bottle.
102 Rinse centrifugation bottles subsequently with 10 to 15 ml LB medium to collect remaining bacteria.
103 Add to bacterial pool and resuspend bacteria by vortexing until homogenous.
104 Distribute bacterial suspension evenly into 8 to 10 50‐ml conical tubes.
  Determine the tare weight before and note on the tube.
105 Spin down the bacteria for 15 min at 6000 × *g*, 4°C.
106 Decant the supernatant if clear; otherwise, spin down again for 10 min at 6000 × *g*, 4°C.
107 Determine the weight of the bacteria pellets in the 50‐ml conical tubes, taking the tare weight into account.
108 Store pellets at −20°C.

### Purification of the STARR‐seq plasmid library

109 Purify one bacterial pellet (maximum, 7.5 g) per column using the Plasmid Plus Giga Kit according to manufacturer's instructions.
110 Elute in 1 ml MonoQ H_2_O and measure concentration.
  Electroporation using the MaxCyte STX transfection system requires the DNA to be suspended in H_2_O; in addition, the DNA concentration needs to be >1 µg/µl.
  Check requirements for other transfection methods or protocols.
111 Elute again in 0.5 ml MonoQ H_2_O.
  If the concentration of the second elution is >2 µg/µl, add to first elution.

### Deep sequencing of the STARR‐seq plasmid library

The quality and complexity of the STARR‐seq plasmid library needs to be assessed by deep sequencing.

*NOTE*: The STARR‐seq plasmid library is used as input for STARR‐seq analysis (see Statistical Analyses).

112 Amplify the STARR‐seq plasmid library using Illumina‐compatible i5 and i7 index primers.
113 Use the STARR‐seq plasmid library diluted to 100 ng/µl (in MonoQ H_2_O) as template for the sequencing‐ready PCR.
114 Prepare the master mix for the sequencing‐ready PCRs (the mix below is for one reaction; scale accordingly):
  1 µl STARR‐seq plasmid library (100 ng/µl)
  2.5 µl Illumina i5 primer (10 µM)
  2.5 µl Illumina i7 primer (10 µM)
  25 µl KAPA 2× HiFi HotStart Ready Mix
  19 µl H_2_O (MonoQ).
  Perform two sequencing ready PCR reactions for focused (BAC) and 10 for genome‐wide libraries.
  The number of PCR reactions depends on the library complexity. Keep in mind to also scale the sequencing depth according to library complexity.
115 Distribute the master mix to PCR strips (50 µl per well).
116 Run the sequencing‐ready PCR:

**Table:** 

1 cycle:	45 s	98°C	(initial denaturation)
9 cycles:	15 s	98°C	(denaturation)
	30 s	65°C	(annealing)
	45 s	72°C	(extension)
1 cycle:	120 s	72°C	(final extension).
117 Add 10 µl of 6× DNA loading dye per 50‐µl PCR reaction.
118 Run 60‐µl sample per well on a 1% agarose gel (see Current Protocols article: Voytas, 2000) at 160 V for 30 min.
  Run 12 µl of 1‐kb DNA ladder for size determination.
  Use SYBR® Safe DNA stain and visualize DNA on a blue light illuminator.
119 Size‐select the amplified STARR‐seq plasmid library (∼1 to 1.5 kb).
  Cut out DNA band (smear) from the gel using blue light illumination.
  Cut‐out size depends on initial library insert size (see steps 12 to 19).
120 Purify size‐selected DNA from two lanes with one QIAquick gel extraction column according to manufacturer's instructions.
  Do not use more than 400 mg agarose per column.
121 Wash twice with 750 µl buffer PE.
122 Elute twice using 50 and then 25 µl EB.
123 Pool eluates from gel extraction step (step 122) and divide into two samples with equal volume.
124 Clean up with two MinElute columns following the Qiagen MinElute PCR purification protocol (according to manufacturer's instructions).
  For focused libraries, use only one column.
125 Elute twice, each time using 10 µl EB per column and pool eluates.
126 Submit ∼500 ng sample for Illumina deep sequencing.
  Final concentration: ∼5‐10 ng/µl.
  Ideally sequence paired‐end. Single‐read sequencing is also possible at the expense of the loss of fragment‐length information (for analysis, reads should be extended by the median size of the STARR‐seq plasmid library (see step 12)).
  IMPORTANT NOTE: The sequenced STARR‐seq plasmid library serves as input for STARR‐seq analysis (peak calling).
127 Store sample at −20°C.

## Basic Protocol 2 MAMMALIAN STARR‐seq SCREENING PROTOCOL

STARR‐seq is a plasmid‐based enhancer‐activity assay that allows the identification of enhancers on a genome‐wide scale and the quantitative assessment of the enhancers’ activities by deep sequencing of reporter transcripts. As for all ectopic assays based on reporter transcript quantification, the STARR‐seq candidate library has to be transfected into the cells of interest, and the reporter transcripts have to be harvested, processed, and sequenced. This protocol describes these steps in human cells for focused and genome‐wide candidate libraries using the hSTARR‐seq_ORI vector (Muerdter et al., 2018). Note that the ORI is used as core promoter; see Background information for details. Library cloning is covered in Basic Protocol 1 (also see Fig. 1, top).

The first step in STARR‐seq is the transfection of the STARR‐seq plasmid library (from Basic Protocol 1) into the cells of interest. The transfected cells are then incubated to allow the transcription of the reporter mRNAs. For human cells, a 6‐hr incubation resulted in the strongest STARR‐seq signal over background (incubation time may vary depending on cell line and transfection method used). After harvesting the cells, total RNA is isolated and the reporter transcripts are enriched as part of the cellular mRNA by oligo(dT)‐based mRNA isolation, followed by Turbo DNase digestion to remove residual plasmids. cDNA synthesis is performed using a reporter‐transcript‐specific reverse‐transcription (RT) primer, and the reporter cDNAs are then selectively amplified by a nested two‐step PCR amplification strategy. To ensure that only reporter cDNA and not residual library plasmids are amplified, we use a primer for the first PCR step (junction PCR) that specifically binds across the splice junction of the reporter transcript. The second PCR step specifically amplifies the candidate sequences flanked by Illumina adapters (Fig. 1, left: see Basic Protocol 1) using Illumina‐compatible index primers. The amplified candidate sequences are then subjected to deep sequencing.

### Materials

Cultured cell line of interest
Appropriate growth medium (for HCT116 or HeLaS3 cells):
DMEM (Gibco cat. no. 52100‐047) containing:
  10% heat‐inactivated FBS (Sigma cat. no. F7524)
  2 mM l‐glutamine (Sigma cat. no. G7513)
STARR‐seq plasmid library (Basic Protocol 1)
Control plasmids, e.g., pIRES‐EGFP (Clontech cat. no. 6029‐1)
1× phosphate‐buffered saline (PBS; Moore, 1996)
1× Trypsin (0.05% trypsin‐EDTA; Gibco cat. no. 25300 054)
Transfection buffer (depends on chosen transfection protocol)
PKR inhibitor (C16 inhibitor) (Sigma cat. no. I9785‐5MG)
TBK1/IKK inhibitors (BX‐795 inhibitor) (Sigma cat. no. SML0694‐5MG)
RNeasy Maxi kit (Qiagen cat. no. 75162)
14.3 M β‐mercaptoethanol (β‐ME) (Sigma cat. no. 63689‐100ML‐F)
70% Ethanol [diluted with DEPC‐treated (see recipe) MonoQ H_2_O]
RNase‐free H_2_O or DEPC‐treated (see recipe) MonoQ H_2_O
RNase Zap (Sigma cat. no. AM9780)
Spike‐in controls (see text over steps 47 to 62)
Dynabeads Oligo‐dT_25_ (Invitrogen cat. no. 61005)
Buffers for Dynabeads Oligo‐dT_25_ (see recipes in Reagents and Solutions):
  2× Binding buffer
  Washing buffer
  Storage buffer
  Reconditioning buffer
10 mM Tris⋅Cl, pH 8.0 and 7.5, RNase‐free (Moore, 1996)
Turbo DNase (Invitrogen cat. no. AM2238) and 10× Turbo DNase buffer
80% Ethanol [diluted with DEPC‐treated (see recipe) MonoQ H_2_O]
RNA CleanXP beads (Beckman Coulter cat. no. A63987)
10 µM dNTP mix (NEB cat. no. 4475)
Primers:
  gene specific RT primer (GSP): CTCATCAATGTATCTTATCATGTCTG
  junction forward (fw) primer: TCGTGAGGCACTGGGCAG*G*T*G*T*C
  junction reverse (rv) primer: CTTATCATGTCTGCTCGA*A*G*C
  * = phosphorothioate bond (protection of primer from 3′ to 5′ exonuclease activity of proof‐reading DNA polymerase; especially important for junction fw primer that specifically binds across the splice junction of the reporter cDNA/transcript)
  Illumina‐compatible i5 and i7 index primers
SuperScript® III reverse transcriptase (supplied with 5× first‐strand buffer and 0.1 M DTT; Invitrogen cat. no. 18080093)
40,000 U/ml Murine RNase Inhibitor (NEB cat. no. M0314L)
AMPure XP beads (Beckman Coulter cat. no. A63882)
RNaseA (Thermo Fisher cat. no. EN0531)
KAPA 2× HiFi HotStart Ready Mix (Kapa Biosystems cat. no KK2601)
EB (10 mM Tris·Cl, pH 8.5)
6× DNA loading dye (see Current Protocols article: Voytas, 2000)
SPRIselect beads (Beckman Coulter cat. no. B23318)

Square plates (24.5 × 24.5–cm; Thermo Scientific cat. no. 166508)
T‐175 or T‐225 culture flasks (Thermo Scientific cat. no. 159910 or 159934)
Automated cell counter
50‐ml conical tubes (e.g., BD Falcon)
Centrifuge (for 50‐ml tubes)
redundant to Thermomixer
Magnetic separator for PCR strips (NEB cat. no. S1515S)
Magnetic separator for 1.5‐ml and 2‐ml tubes (Thermo Fisher cat. no. 12321D)
Magnetic separator for 5‐ml tubes (Thermo Fisher cat. no. 12303D)
Magnetic separator for 15‐ml tubes (Thermo Fisher cat. no. 12301D)
Tissue Ruptor (Qiagen cat. no. 9002756)
Tissue Ruptor disposable probes (Qiagen cat. no. 990890)
15‐ml conical/polystyrene tubes (Thermo Fisher cat. no. 05‐527‐90)
1.5‐ml DNA LoBind tubes, RNase‐free (Eppendorf cat. no. 022431048)
5‐ml tubes, RNase‐free (Eppendorf cat.no. 0030119487)
Rolling shaker
Thermomixer
PCR strips (Biozym cat. no. 711030)
PCR 8er‐CapStrips (Biozym cat. no. 711040)
Thermal cycler (PCR machine)

Additional reagents and equipment for transfection (see Support Protocol, references cited under step 1, below, and other relevant articles in Current Protocols in Molecular Biology), fluorescence‐activated cell sorting (FACS; Robinson et al., 2019), RNA and DNA quantitation (see Current Protocols article: Gallagher & Desjardins, 2006), DNA sequencing (see Current Protocols article: Shendure, Porreca, & Church, 2008), and agarose gel electrophoresis (see Current Protocols article: Voytas, 2000)

### Transfection of human cells with the STARR‐seq plasmid library

#### Establishing transfection conditions for human cells

1 Select a suitable transfection method for the cell line of interest that yields high transfection efficiencies and makes it possible to scale to the required throughput.
  We recommend using electroporation for transfection due to scalability and efficiency (see Support Protocol). Other transient transfection methods like chemical transfection have been successfully used for STARR‐seq (Barakat et al., 2018; Vockley et al., 2016).
  Viral transfection (infection) has been used for other MPRAs (Inoue et al., 2017; Maricque et al., 2017; Nguyen et al., 2016; Shen et al., 2016); however, not for STARR‐seq. We anticipate that viral infection should be compatible with STARR‐seq as well. This would require the construction of a virus‐compatible STARR‐seq plasmid.
2 Test and optimize the transfection efficiency of the cell line of interest by transfecting control plasmids that strongly express a fluorescence protein (e.g., GFP).
  We recommend using pIRES‐EGFP (Clontech cat. no. 6029‐1).
  Determine the transfection efficiency using FACS (Robinson et al., 2019).
  The transfection efficiency should be as high as possible, ideally >60% for genome‐wide screens to account for the high library complexity and to ensure high‐quality screens (high library coverage).
3 Determine if the cell line of interest is generating a type‐I interferon (INF) response upon plasmid transfection.
  Consult the literature (e.g., Muerdter et al., 2018; supplementary Fig. 2) or test the upregulation of INF‐responsive genes such as IFIT2, MX1, OAS3, or ISG15 after plasmid transfection (24 and 48 hr post transfection) using qPCR (see Muerdter et al., 2018).
  If an interferon response is expected, use signaling pathway inhibitors to prevent false‐positive and false‐negative STARR‐seq signals (enhancer activities) (see Strategic Planning and Background Information).

**Figure 2:**
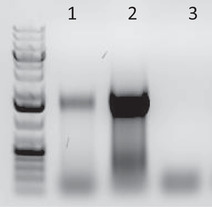
Test PCR on 1% agarose gel (lanes): left lane, Fermentas GeneRuler 1 kb plus; (1) 10 µl of sequencing‐ready PCR with five cycles; (2) 10 µl of sequencing‐ready PCR with nine cycles; and (3) 10 µl of sequencing‐ready PCR with nine cycles of minus RT.

#### Transfection of human cells

We provide two variants of the transfection protocol: (1) a general protocol for transfection of human cells with the STARR‐seq plasmid library (see below) and (2) an optimized transfection protocol for human cells using the electroporation device “MaxCyte STX scalable transfection system” (Support Protocol).

4 Seed/split cells 24 hr before transfection in square plates (24.5 × 24.5 cm).
  Cells should be 80% confluent on transfection day for optimal transfection efficiency. An 80% confluent square plate contains around 8 × 10^7^ cells HCT116.
  Use 4 × 10^8^ cells per replicate for a genome‐wide screen (five to six square plates) or 8 × 10^7^ cells for a focused screen (one to two square plates). Note that these cell numbers were determined in HCT116 and HeLa S3 cells using electroporation (transfection efficiency >80%, viability >90%). The number of cells may vary, depending on transfection method, cell line, transfection efficiency, cell viability, and STARR‐seq plasmid library complexity. The number of cells needs to be determined prior to transfection of the STARR‐seq plasmid library for every cell line.
  Perform at least two independent replicates per screen. To monitor the transfection efficiency for each STARR‐seq screen, always include a transfection control (e.g., transfect a small number of cells using a GFP expressing plasmid, e.g., pIRES‐EGFP). Measure efficiency by FACS (Robinson et al., 2019).
5 Harvest cells after 24 hr for transfection.
6 Remove the growth medium completely.
7 Wash cells carefully with 12 ml of 1 × PBS. Remove 1 × PBS completely.
8 Add 8 ml of 1× trypsin per square plate (cells need to be covered completely) and incubate at 37°C for ∼5 min.
  Steps 8 and 9 are only needed for adherent cells.
9 Add 12 ml growth medium to stop the action of trypsin, resuspending the cells by pipetting up and down thoroughly.
10 Pool cells from all square plates in a T‐175 or a T‐225 flask, depending on total volume, and mix thoroughly.
11 Count the pooled cells using an automated cell counter.
  Use 4 × 10^8^ cells for a genome‐wide screen or 8 × 10^7^ cells for a focused screen. The number of cells may vary depending on transfection protocol and cell line.
12 Transfer the required number of cells to 50‐ml conical tubes.
  Use cell count from step 11 to calculate the volume of cell suspension needed.
13 Spin down cells 5 min at 125 × *g*, room temperature.
14 Remove medium and resuspend each cell pellet in 5 ml transfection buffer, then pool all resuspended pellets in a 50 ml conical tube.
  The transfection buffer depends on transfection method/protocol.
15 Spin down cells 5 min at 125 × *g*, room temperature.
16 Resuspend cells in appropriate transfection buffer.
  The transfection buffer as well as the volume depend on transfection method/protocol.
17 Transfect cells with the STARR‐seq plasmid library using the previously determined transfection method according to manufacturer's instructions and previously determined conditions.
  Transfect 20 µg STARR‐seq plasmid library per 1 × 10^7^ cells.
18 Resuspend transfected cells in growth medium and plate 50 ml per square plate (final concentration: 1 × 10^6^ cells/ml).
  For cells with an interferon (INF) response, add C16 and BX‐795 inhibitors to the transfected cells at a final concentration of 1 µM per inhibitor (add 40 µl of 1 mM stock solution to 40 ml growth medium).
19 Incubate at 37°C for 6 hr.
  The incubation time of STARR‐seq is counted from plating the cells (step 18) until cell lysis for total RNA isolation (step 33).
  Using electroporation, we found 6 hr to be ideal for STARR‐seq in several human cell types (determined by the signal over background ratio). The optimal incubation time of STARR‐seq may depend on the transfection method and cell line, and should be determined individually.
  If using lipofection, the incubation time should be extended to 12 to 24 hr. The optimal time frame needs to be determined experimentally.

### Harvesting cells transfected with STARR‐seq plasmid library

20 Harvest cells to ensure cell lysis 6 hr post transfection when using electroporation.
  If using adherent cells: 30% to 40% of the cells are not yet attached after 5 to 6 hr post transfection. Therefore, non‐attached cells from the growth medium also need to be harvested.
  If performing a genome‐wide screen, start harvesting of cells after ∼5 hr to ensure cell lysis after 6 hr.
21 Transfer 50 ml growth medium (from one square plate) to two 50‐ml conical tubes.
  The growth medium contains transfected cells that have not attached to the culture flask yet.
22 Wash the cells with 10 ml of 1 × PBS.
23 Transfer the PBS wash to the 50‐ml Falcon tubes from step 21, for maximum recovery.
24 Add 8 ml 1× trypsin per square plate and incubate at 37°C for 4 to 5 min.
25 During trypsin incubation, spin down growth medium from step 21 for 5 min at 125 to 300 × *g*, room temperature, and aspirate supernatant.
26 Add 12 ml serum‐containing growth medium per square plate to stop the action of trypsin, resuspending the cells by pipetting up and down thoroughly.
27 Combine this cell suspension with the cell pellet from step 21.
28 Spin down the cells 5 min at 125 to 300 × *g*, room temperature.
29 Wash cells with 20 ml of 1 × PBS.
30 Spin cells down 5 min at 125 to 300 × *g*, room temperature.
31 Remove PBS by aspiration.
  Leave 200 to 400 µl of 1× PBS (∼1 to 2 mm) covering the cell pellet in the 50‐ml conical tube.
  Resuspend the cells in 200 to 400 µl of 1× PBS by softly flicking the tube prior to cell lysis (step 32).

### Total RNA isolation

Perform total RNA isolation using the Qiagen RNeasy Maxi Kit. Read the manufacturer's instructions before use. Clean and wipe fume hood and all equipment with RNase Zap (or similar) to ensure an RNase‐free working environment.

Prepare the following reagents freshly before starting (the indicated volumes refer to one sample). Calculate the total volume of the reagents by multiplying the total number of samples plus one (e.g., 4 samples +1 = 5) with the reagent volume per sample.

RLT buffer (15 ml): Add 150 µl 14.3 M β‐ME to 15 ml RLT buffer (contained in RNeasy Maxi Kit). Work in an RNase‐free fume hood to avoid β‐ME inhalation.
70% ethanol (15 ml): Dilute 10.5 ml 100% ethanol with 4.5 ml RNase‐free/DEPC‐treated MonoQ H_2_O
RPE buffer (contained in the RNeasy Maxi Kit as concentrate). Add four volumes of 100% ethanol to the RPE buffer concentrate (volumes are indicated on the bottle) to obtain the working solution.
Prepare the Qiagen TissueRuptor and disposable probes.
Install a waste container in the fume hood to dispose of β‐ME‐contaminated plastics and solutions.

32 Resuspend the cell pellet in the 50‐ml conical tube by vortexing at medium speed.
  This is important to ensure complete and efficient disruption/lysis of cells.
33 Lyse the cells by adding 15 ml RLT buffer (+β‐ME) to the resuspended cell pellet.
  First add ∼5 ml RLT (+β‐ME) dropwise while vortexing the cells, then add the remaining RLT (+β‐ME), close the tube, and vortex until the solution is homogeneous (5 to 10 s).
  Repeat steps 32 and 33 for all samples/cell pellets.
34 Homogenize the cells using the Qiagen TissueRuptor with a disposable probe at full speed.
  Homogenize each cell pellet/sample for 4.5 min while constantly moving the disposable probe within the tube. Note that shorter homogenization times lead to reduced RNA yield.
  Repeat step 34 for all samples. To avoid cross‐contamination of samples, use a fresh disposable probe for each sample (meaning a different STARR‐seq plasmid library or cell line).
  The disposable probe can be re‐used for cell pellets of the same sample (genome‐wide screen).
35 Add 15 ml 70% ethanol to the homogenized lysate, then mix thoroughly by shaking vigorously for 20 s.
36 Transfer 15 ml of lysate to an RNeasy Maxi column (maximum volume, 15 ml).
  Use one RNeasy Maxi column per cell pellet (8 × 10^7^) of a focused screen and two columns for the cell pellet of a genome‐wide screen (2 × 10^8^ cells per column). Do not overload the column, as this results in reduced RNA yield. Maximum capacity may vary between different cell lines.
37 Centrifuge 5 min at >3200 × *g*, 25°C, and discard flow‐through.
38 Repeat steps 36 and 37 with the remaining 15 ml lysate and discard flow‐through.
39 Add 15 ml RW1 buffer (from the RNeasy kit), centrifuge for 5 min at >3200 × *g*, 25°C, then discard the flow‐through.
40 Add 10 ml RPE buffer prepared as described in the text above step 32, centrifuge 2 min at >3200 × *g*, 25°C, and discard the flow‐through.
41 Add 10 ml RPE buffer prepared as above, then centrifuge for 10 min at >3200 × *g*, 25°C
  The extended centrifugation time (10 min) is necessary to dry the membrane and remove residual wash buffer.
42 Transfer the column to a new collection tube (provided in the kit) for elution.
43 To elute, add RNase‐free H_2_O directly to the silica‐gel membrane and incubate for 2 min before centrifuging for 5 min at >3200 × *g*, 25°C.
44 Elute three times subsequently with the following volumes: first in 1.2 ml, second in 1 ml, third in 0.5 ml.
  Subsequent elution steps are important for maximum recovery of total RNA. If the concentration is expected to be low, e.g., for focused screens, elute in 0.5‐ml steps only. If the concentration after the third elution is >300 ng/µl, elute further in 0.5‐ml steps. If using more than one column per sample (typically for genome‐wide screens), pool the same elution fractions to obtain pools of the first, second, and third elution steps.
45 Measure the RNA concentration (see Current Protocols article: Gallagher & Desjardins, 2006) of the pooled elution fractions.
46 Pool elution fractions to obtain a final concentration of ≤750 ng/µl and a volume matching full or half milliliters (to facilitate handling during mRNA isolation in subsequent steps).
  750 ng/µl is the maximum concentration you can use in the next step due to the binding capacity of the Oligo‐dT_25_ beads.
  If processing more than one sample in parallel, adjust all samples to the same volume, to facilitate handling during mRNA isolation.
  Samples can be stored at −80°C; save 5 µl for gel analysis to determine RNA integrity.
  The maximum volume of the sample for 5‐ml tubes is 2 ml, for 15‐ml tubes, 5 ml.

### Optional: Spike‐in control for library normalization

Spike‐in controls are highly recommended if different conditions are examined using STARR‐seq or global changes in enhancer activities are expected (e.g., the induction or inactivation of many or all enhancers). Spike‐in controls are used to normalize different STARR‐seq screens to perform comparative analyses.

Total RNA from a previous screen of individual enhancers or a focused library can be used for spike‐in controls—the candidates for the spike‐in library must be different from the candidates for the current screen (e.g., use a different BAC region or DNA from a different closely related species). This ensures that the STARR‐seq and spike‐in transcripts are processed simultaneously throughout all steps of the protocol. We prepare spike‐in controls beforehand and keep aliquots of total RNA. “Spike‐in (total) RNA” is added to the total RNA of the STARR‐seq screens.

We recommend two variants of spike‐in controls:

- Pool of individual enhancers (steps 47 to 55).
- Genomic regions covered by BACs containing multiple enhancers (steps 56 to 62).

Use spike‐in control (1) from steps 42 to 50 or (2) from steps 56 to 62.

#### Pool of individual enhancers

47 Identify putative enhancers from closely related species that can be mapped unambiguously to only the respective reference genome to avoid cross mapping between STARR‐seq and spike‐in reads.
  For human cells, e.g., mouse enhancers can be used.
48 Clone individual putative enhancers flanked by the Illumina adapters into the STARR‐seq screening plasmid.
  For the cloning protocol, see Basic Protocol 1—STARR‐seq plasmid library cloning
  Recommended, >10 to 15 enhancers.
49 Verify the successful cloning by Sanger sequencing (Shendure et al., 2008).
50 Mix spike‐in control plasmids at an equimolar ratio.
51 Transfect spike‐in control plasmid mix to the same cell line that is used for STARR‐seq.
52 Extract total RNA from the transfected cells (see steps 32 to 46—total RNA isolation procedure).
53 Dilute the spike‐in total RNA to 100 ng/µl with RNase‐free MonoQ H_2_O and aliquot amounts needed per one genome‐wide screen.
  Avoid repeated freeze‐thaw cycles.
  Store spike‐in total RNA at −80°C.
54 Add an appropriate amount (ratio) of spike‐in to focused or genome‐wide STARR‐seq screens.
  The ratio of spike‐in to STARR‐seq total RNA depends on the size, i.e., complexity of the STARR‐seq plasmid library (ratios successfully tested: focused library: 1:10,000 or genome‐wide: 1:1000).
55 Add spike‐in total RNA to STARR‐seq total RNA after its concentration has been adjusted prior to mRNA isolation (step 46).
  The ratio of spike‐in to STARR‐seq total RNA needs to be experimentally determined by testing different ratios (e.g., 1:100, 1:1000, 1:10,000) in a standard STARR‐seq screen. Spike‐in reads should be in the range of ∼0.02% of total reads.

#### Genomic regions covered by BACs containing multiple enhancers

56 Identify putative enhancers from closely related species.
57 Identify BACs that cover genomic regions containing single/multiple enhancers.
58 Perform STARR‐seq library cloning protocol (Basic Protocol 1) using BAC DNA from step 57.
59 Transfect spike‐in control library to the same cell line used for STARR‐seq.
60 Continue as described above (see step 52).
61 Process a fraction of the spike‐in control screen to confirm successful performance of STARR‐seq using the spike‐in control library. Make sure that the control enhancers are detected after deep sequencing.
62 Test the ratio of spike‐in to STARR‐seq total RNA (see step 55).

### mRNA/poly(A)^+^ RNA isolation with Dynabeads Oligo (dT)_25_

Use 2 vol of Dynabeads Oligo (dT)_25_ for 1 vol of total RNA, e.g., 2 ml of Dynabeads Oligo (dT)_25_ per 1 ml of total RNA.

Prepare before starting:

2× binding buffer (quantity 2.5× the starting volume of beads)
Washing buffer (quantity 2× the starting volume of beads)
Storage buffer (quantity 5× the starting volume of beads)
Reconditioning buffer (quantity 3× the starting volume of beads).

All buffers need to be at room temperature before use.

#### Prepare RNA for binding to Dynabeads Oligo (dT)_25_

63 Heat total RNA to 65°C in a heating block.
  Incubate the RNA for 12 min when using 5‐ml or 15‐ml tubes (genome‐wide screen); incubate for 7 min when using 1.5‐ml tubes (focused screen).
64 Place total RNA on ice immediately for 5 or 3 min.
65 Incubate total RNA at room temperature for 1 min.

#### Washing procedure for Dynabeads Oligo (dT)_25_

This step prepares the beads for binding to the mRNA. Resuspend Dynabeads Oligo (dT)_25_ beads thoroughly by vortexing before use.

66 Transfer the beads to an appropriate tube.
  Use 2 vol of Dynabeads Oligo (dT)_25_ for 1 vol of total RNA.
67 Place the tube on a suitable magnetic separator and incubate until the solution is clear.
68 Remove the storage buffer completely from the beads.
69 Wash beads twice with 1 vol (volume refers to starting volume of beads) of 2× binding buffer. Vortex thoroughly, then place on magnetic separator for 1.5 min (until solution is clear).
70 Resuspend beads in ½ vol of 2× binding buffer (referring to starting volume of beads).

#### Oligo‐dT purification of mRNA using Dynabeads Oligo (dT)_25_

71 Add 1 × vol of total RNA (from step 65) to 1 vol of beads (resuspended in 2× binding buffer) to obtain a final concentration of 1× binding buffer.
  Mix gently but thoroughly by carefully vortexing or pipetting up and down.
72 Incubate at room temperature for 10 min under constant rotation using a rolling shaker. To collect all beads from the lid, gently quick‐spin tubes at <100 × g for ∼5 s.
73 Place the tube on the magnetic separator for 2 min and completely remove the supernatant.
74 Wash the beads twice carefully using 1 vol washing buffer (volume corresponds to the starting volume of the beads). To resuspend the beads, gently invert the tube. Place the tube on the magnetic separator for 2 min and completely remove the supernatant.
  IMPORTANT NOTE: Do not vortex while mRNA is bound to the beads!
75 Remove the remaining washing buffer completely before elution. Spin tubes at <100 × g for 5‐10 s. Place on the magnetic separator and remove the remaining liquid completely.
  This is important to avoid contamination of the eluate with washing buffer.
76 Elute in 50 µl 10 mM Tris·Cl (pH 7.5) per 1 ml of beads (starting volume). Mix by pipetting up and down 10 times. Incubate at 80°C for 3 min in a thermomixer while shaking (750 rpm).
77 Immediately transfer the tubes to the magnetic separator and incubate for >1 min.
  The immediate transfer to the magnet is essential to avoid re‐annealing of mRNA to Oligo‐dT beads.
78 Transfer the eluted mRNA (supernatant) to a new RNase‐free 1.5 ml LoBind tube.
  The 1.5‐ml LoBind tubes are RNase‐free as purchased from the manufacturer.
79 Elute again with 25 µl 10 mM Tris·Cl (pH 7.5) per 1 ml beads (starting volume). Repeat steps 76 to 78.
80 Combine all first and second elutions of the same sample.
81 Measure RNA concentration of the final mRNA pool (see Current Protocols article: Gallagher & Desjardins, 2006). Save 200 ng mRNA for gel analysis
82 Resuspend the beads in 1 vol storage buffer (same volume as starting volume) and store at 4°C.
  Dynabeads Oligo (dT)_25_ can be regenerated (see Reagents and Solutions for details) and re‐used.

### Turbo DNase digest

83 Prepare Turbo DNase reaction master mix as follows (see steps 84 to 87 for mixing procedure; quantities are for one reaction):
  88 µl mRNA (<200 ng/µl)
  10 µl 10× Turbo DNase buffer
  2.4 µl Turbo DNase.
  Process the entire mRNA and scale the number of reactions accordingly. Perform at least two Turbo DNase reactions per sample (applies to focused screen).
84 Combine mRNA and 10× Turbo DNase buffer.
85 Mix thoroughly by vortexing or pipetting.
86 Add Turbo DNase to the master mix.
  As Turbo DNase is sensitive to mechanical stress, avoid extensive vortexing.
87 Invert the tube gently and mix by slowly pipetting up and down 20 times.
88 Distribute the master mix into PCR strips (100 µl master mix per well).
89 Incubate for 30 min at 37°C in a thermal cycler with the heated lid set to 65°C, then hold at 4°C.

### Turbo DNase reaction cleanup with RNA CleanXP beads

Before starting:

Prepare 80% ethanol (diluted with RNase‐free/DEPC‐treated MonoQ H_2_O)
Warm RNA CleanXP beads to room temperature.

90 Combine two Turbo DNase reactions per sample (2 × 100 µl = 200 µl final volume).
91 Add 1.8 vol beads to 1 vol mRNA (add 360 µl beads to 200 µl mRNA) using RNase‐free 1.5‐ml LoBind tubes.
  Resuspend RNA CleanXP beads thoroughly by vortexing before use.
92 Mix thoroughly by vortexing briefly and pipetting up and down 20 times.
93 Incubate for 15 min at room temperature.
94 Transfer to magnetic separator and incubate for 10 min at room temperature.
  The solution needs to be clear—allow beads to migrate to the magnet.
  Keep tubes/samples on magnetic separator for the following steps (except elution); always pipette onto the tube wall opposite the magnet.
95 Remove all liquid.
96 Wash the beads twice on the magnet, each time with 1 ml of 80% ethanol, and incubate for 2 min.
  Make sure that beads are covered completely with 80% ethanol.
97 After the second wash, completely remove the ethanol.
98 Dry the beads at room temperature for 5 to 10 min, allowing the evaporation of any remaining 80% ethanol.
  Keep tubes on magnet with open lids. Avoid over‐drying and elute as soon as the bead pellet starts to show cracks.
99 To elute, add 20 µl RNase‐free H_2_O per Turbo DNase reaction (40 µl per tube).
  Close the tube and remove from magnet.
100 Resuspend the beads completely by vortexing thoroughly and incubate at 37°C on a thermomixer for 3 min while shaking (300 rpm).
101 Transfer the beads/tubes immediately onto the magnetic separator and incubate for 1 min.
  Carefully tilt magnet to allow the beads to migrate upwards on wall.
102 Transfer mRNA (supernatant) to a new RNase‐free 1.5‐ml LoBind tube and measure the mRNA concentration.
  Pool all eluted mRNA of the same screen/sample. Save 200 ng of pooled mRNA for gel analysis.

### Reverse transcription (RT)/first‐strand cDNA synthesis

Using a reporter transcript–specific RT primer (GSP) allows processing of up to 4 to 5 µg mRNA per RT reaction. To determine the number of RT reactions, divide the RNA concentration by 5 and round this number up to the nearest multiple of 5 (e.g., 4 becomes 5; 8 becomes 10). This is the total number of RT reactions per sample

103 Prepare RT master mix I (MMI). For one reaction:
  X µl mRNA (divide total volume of mRNA by total number of RT reactions)
  1 µl dNTP mix (10 µM)
  1 µl GSP (2 µM) (gene‐specific RT primer; CTCATCAATGTATCTTATCATGTCTG)
  Make up to 13 µl with RNase‐free H_2_O.
  Process five RT reactions per tube (65 µl total volume).
  Perform 1 minus RT control (replace SSIII with H_2_O).
104 Distribute 65 µl of RT master mix I per well to PCR strip (corresponding to 5 reactions/well).
105 Incubate at 65°C for 5 min in a thermal cycler, then transfer to ice for 1 min.
106 Prepare RT master mix II (mix thoroughly). For one reaction:
  4 µl 5× first‐strand buffer
  1 µl DTT (0.1 M)
  1 µl RNase Inhibitor (40,000 U/ml)
  1 µl SuperScript III (H_2_O for minus RT control)
  Use same number of reactions as in step 103.
107 Add 35 µl RT reaction mix II to 65 µl RT reaction mixture I (5 reaction/well: final volume: 100 µl).
108 Add 7 µl minus RT reaction mix II to 13 µl RT reaction mix I.
109 Incubate in thermal cycler at 50°C for 1 hr, then 70°C for 15 min, and hold at 4°C, to prepare the cDNA.
  Optional: samples can be stored at −20°C.

### RNaseA treatment

CAUTION: Avoid pipetting RNaseA next to RNase‐free working space.

110 Add 1 µl RNaseA (10 mg/ml) per five RT reactions (100 µl).
111 Add 0.2 µl RNaseA per minus RT reaction.
112 Incubate for 1 hr at 37°C in thermal cycler.
  Optional: Samples can be stored at −20°C

### cDNA purification with AMPure XP beads

Before starting:

Prepare 80% ethanol (diluted with RNase‐free/DEPC‐treated MonoQ H_2_O)
Warm AMPureXP beads to room temperature
Resuspend AMPureXP beads thoroughly by vortexing before use.

113 Pool all cDNA samples, mix them thoroughly, and distribute 200 µl (= 10 RT reactions) into 1.5‐ml LoBind tubes.
  Process minus RT reaction separately.
114 Add 1.8 vol AMPureXP beads to 1 vol cDNA (add 360 µl beads to 200 µl cDNA; 36 µl to minus RT) in 1.5‐ml LoBind tubes.
115 Mix thoroughly by vortexing briefly and pipetting up and down 20 times.
116 Incubate for 15 min at room temperature.
117 Transfer to magnetic separator and incubate 10 min at room temperature.
  Keep tubes/samples on magnetic separator for the following steps (except elution), pipette on opposite tube wall.
118 Remove all liquid.
119 Wash the beads twice with 1 ml 80% ethanol while on magnet, incubating 2 min each time.
  Make sure that beads are covered completely.
120 After the second wash, completely remove the ethanol.
121 Dry the beads at room temperature for 5 to 10 min, allowing the evaporation of any remaining ethanol.
  Keep tubes on magnet with open lids. Avoid over‐drying and elute as soon as the bead pellet starts to show crack.
122 To elute, add 20 µl H_2_O per RT reaction (100 µl per tube). Close the tube and remove from the magnet.
123 Resuspend the beads completely by vortexing thoroughly, and incubate at 37°C on a thermomixer for 3 min while shaking (300 rpm).
124 Transfer the beads/tubes immediately onto the magnetic separator and incubate for 1 min.
  Carefully tilt magnet to allow beads to migrate upwards on wall.
125 Transfer cDNA (supernatant) to a new RNase‐free 1.5‐ml LoBind tube.
126 Pool all cleaned‐up cDNA samples (except minus RT).
  For the subsequent steps (step 127 onwards; step 144 onwards), the template for each PCR reaction is the equivalent of one RT reaction (1 RT reaction/4 to 5 µg mRNA = 1 PCR reaction).

### Junction PCR (jPCR)

The number of junction PCR (jPCR) reactions corresponds to the number of RT reactions.

Each cleaned‐up RT reaction/cDNA sample (4 to 5 µg mRNA) serves as template for one jPCR reaction.

The jPCR specifically enriches reporter transcripts and disfavors the candidate library plasmids, as the forward primer binds only to the spliced intron of the reporter transcript.

The complexity of the reporter transcript pool linearly correlates with amount of cDNA amplified.

For maximum complexity/coverage, amplify all of the cDNA during the junction PCR.

*CAUTION*: When processing more than one STARR‐seq screen in parallel, work extremely carefully to avoid cross contamination of screens. Ideally separate screens physically (e.g., work on different benches, use different pipets, regularly change gloves).

127 Prepare the junction PCR (jPCR) master mix. For one reaction:
  20 µl cDNA (= one RT reaction)
  25 µl KAPA 2× HiFi HotStart Ready Mix
  2.5 µl junction fw primer (10 µM)
  2.5 µl junction rev primer (10 µM),
  Scale according to the number of RT reactions, i.e., perform the same number of jPCR reactions as RT reactions. Use the cDNA from one RT reaction as template for one junction PCR reaction.
128 Vortex and briefly spin down.
129 Distribute 50 µl master mix per well into PCR strips.
130 Run the following PCR program:

**Table:** 

1 cycle:	45 s	98°C	(initial denaturation)
16 cycles:	15 s	98°C	(denaturation)
	30 s	65°C	(annealing)
	70 s	72°C	(extension)
1 cycle:	120 s	72°C	(final extension).
  The extension time depends on the candidate library insert size (here 1.2 to 1.5 kb). Adjust the extension time according to candidate library insert size.

### jPCR purification with AMPure XP beads

Before starting:

Prepare 80% ethanol (diluted with MonoQ H_2_O)
Warm AMPureXP beads to room temperature
Resuspend AMPureXP beads thoroughly by vortexing before use.

131 Pool all jPCR reactions (except minus RT), mix thoroughly, and distribute 250 µl (=five RT reactions; keep minus RT separate) into 1.5‐ml LoBind tubes.
132 Add 0.8 vol beads to 1 vol of PCR reaction (add 200 µl beads to 250 µl PCR reaction; 40 µl to minus RT) in a 1.5‐ml LoBind tube, briefly vortex, then pipette up and down 20× to mix.
133 Incubate 15 min at room temperature.
134 Transfer to magnetic separator and incubate 10 min at room temperature.
  Keep tubes/samples on magnetic separator for the following steps (except elution); pipette onto opposite tube wall.
135 Remove all liquid.
136 Wash twice with 1 ml 80% ethanol, incubating 2 min (while on magnet) for each wash.
  Make sure that beads are covered completely.
137 After the second wash, make sure to remove ethanol completely.
138 Dry beads at room temperature to allow evaporation of remaining 80% ethanol for 5 to 10 min.
  Keep tubes on magnet with open lids.
  Avoid over‐drying and elute as soon as the bead pellet starts to show cracks.
139 To elute, add 20 µl EB per jPCR reaction (100 µl per tube), close the tube, and remove from magnet.
140 Resuspend beads completely by vortexing thoroughly and incubate at 37°C on thermomixer for 3 min while shaking (300 rpm).
141 Transfer beads/tubes immediately onto magnetic separator and carefully tilt magnet to allow the beads to migrate upwards on wall. Incubate for 1 min.
142 Transfer supernatant to new tube.
143 Pool all cleaned‐up jPCR reactions (except minus RT)

### Sequencing‐ready PCR

The sequencing‐ready PCR is the final amplification step, preparing the STARR‐seq screens for Illumina sequencing.

First, perform a test PCR to determine how many PCR cycles (usually between five and nine) are necessary to amplify STARR‐seq transcripts to a level that provides sufficient material for deep sequencing, while avoiding overamplification. Do not use <5 cycles for the PCR amplification of the STARR‐seq cDNA. The minus RT control should be amplified with the highest number of cycles used during the test PCR. The minus RT reaction controls for plasmid DNA contamination of the STARR‐seq reporter cDNA and should not result in a PCR product.

### Test PCR

144 Prepare the test PCR mix:
  20 µl jPCR (from pool) or 20 µl minus RT
  2.5 µl Illumina i5 primer (10 µM)
  2.5 µl Illumina i7 primer (10 µM)
  25 µl KAPA 2× HiFi HotStart Ready Mix.
145 Amplify the jPCR product with five and nine cycles using the following PCR program:

**Table:** 

1 cycle:	45 s	98°C	(initial denaturation)
5 or 9 cycles:	15 s	98°C	(denaturation)
	30 s	65°C	(annealing)
	45 s	72°C	(extension)
1 cycle:	120 s	72°C	(final extension).
146 Amplify the minus RT reaction with nine cycles only.

### Gel analysis

147 Perform gel electrophoresis of the test PCR using a 1% agarose gel (well size ∼15 µl) at 145 V for 15 min (Voytas, 2000).
148 Load 10 µl of each test PCR mixed with 2 µl 6× DNA loading dye.
149 Determine the optimal number of PCR cycles by band intensity of the PCR product after five and nine PCR cycles on the 1% agarose gel (see Fig. 2).
  For the example in Figure 2, a faint band/smear is observed around 1.2 to 1.5 kb after five cycles and overamplification after nine cycles (in this case use five cycles to amplify the jPCR products).
  If five cycles already result in overamplification, reduce the amount of template (jPCR product) per sequencing‐ready PCR reaction. Use 5 µl template and repeat test PCR.
  Do not reduce the number of cycles for the junction PCR to compensate!
  If you do not detect a band after nine cycles, repeat the test PCR with 12 cycles. A higher number of cycles may indicate low complexity of the reporter transcript pool (e.g., due to poor performance of the transfection or RNA processing). A high number of PCR cycles (>10 to 15) increases the risk of PCR duplications and may lead to poor STARR‐seq signals.

### Sequencing‐ready PCR

Use the number of PCR cycles which was determined via “test PCR” (step 144 to 149). Perform two sequencing‐ready PCRs for focused (BAC) and 10 to 20 for genome‐wide screens. Use different Illumina index primers for each STARR‐seq screen to allow multiplexed deep sequencing.

150 Prepare the sequencing‐ready master PCR mix (scale accordingly):
  20 µl jPCR product
  2.5 µl Illumina i5 primer (10 µM)
  2.5 µl Illumina i7 primer (10 µM)
  25 µl KAPA 2×HiFi HotStart Ready Mix.
151 Distribute the master mix to PCR strips (50 µl per well) and run the sequencing‐ready PCR:

**Table:** 

1 cycle:	45 s	98°C	(initial denaturation)
*x* cycles*:	15 s	98°C	(denaturation)
	30 s	65°C	(annealing)
	45 s	72°C	(extension)
1 cycle:	120 sec	72°C	(final extension).

^*^Number of cycles as determined in test PCR.

### Sequencing‐ready PCR purification with SPRIselect beads

Before starting:

Prepare 80% ethanol (diluted with MonoQ H_2_O)
Warm SPRIselect beads to room temperature
Resuspend SPRIselect beads thoroughly by vortexing before use

It is important to use SPRIselect beads, to allow the selective purification of DNA >800‐1000 bp.

**Figure 3:**
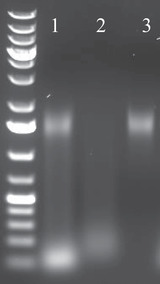
Sequencing‐ready PCR on 1% agarose gel (lanes): left lane, Fermentas GeneRuler 1 kb plus; (1) 10 µl of the pooled PCRs; (2) 10 µl of the supernatant, taken after PCR has bound to the beads; and (3) 250 ng cleaned‐up PCR product.

152 Pool all sequencing‐ready PCR reactions, mix thoroughly, and distribute 100 µl (= 2 reactions) for focused or 500 µl (= 10 reactions) for genome‐wide screens to 1.5‐ml LoBind tubes.
153 Add 0.5 vol SPRIselect beads to 1 vol of PCR reaction (add 250 µl beads to 500 µl PCR reaction) in 1.5‐ml LoBind tubes, briefly vortex, and pipette up and down 20 times to mix.
  It is critical to precisely mix the exact volumes of SPRIselect beads and PCR reaction at a ratio of 0.5 (beads/PCR), as the beads do not bind DNA at ratios lower than 0.5. Using a too low a volume of beads leads to loss of the sample.
  A ratio of 0.5 is used to specifically purify the sequencing‐ready PCR products and exclude all nonspecific products that are smaller than 1000 bp.
154 Incubate 10 min at room temperature.
155 Transfer tubes to magnetic separator and incubate 10 min at room temperature.
  Keep tubes/samples on magnetic separator for the following steps (except elution); pipette onto opposite tube wall.
156 Transfer entire supernatant to a new tube.
  Keep supernatant until successful purification is confirmed; in case the PCR products failed to bind to the beads, they can be re‐extracted from the supernatant.
157 Wash beads twice with 1 ml 80% ethanol, incubating 2 min (while on magnet) each time.
  Make sure that beads are covered completely.
158 After the second wash, make sure to remove 80% ethanol completely.
159 Dry beads at room temperature to allow evaporation of remaining 80% ethanol for 2 to 5 min.
  Keep tubes on magnet with open lids. Avoid over‐drying and elute as soon as the bead pellet starts to show cracks.
160 To elute, add 10 µl nuclease‐free H_2_O per PCR reaction (two reactions: 20 µl, 10 reactions: 100 µl), close the tube, and remove from magnet.
161 Resuspend the beads completely by vortexing thoroughly and incubate at 37°C on thermomixer for 3 min while shaking (300 rpm).
  Transfer beads/tubes immediately onto magnetic separator and carefully tilt magnet to allow the beads to migrate upwards on wall. Incubate for 1 min.
162 Transfer supernatant/sample to a new tube.
163 Pool all purified sequencing‐ready PCR samples.
164 Measure the DNA concentration (see Current Protocols article: Gallagher & Desjardins, 2006).
165 Check the successful purification of the sequencing‐ready PCR products on a 1% agarose gel (see Fig. 3). Also see Current Protocols article: Voytas (2000)
  Load 10 µl pooled PCR product (unpurified), 10 µl of the supernatant, and 250 ng cleaned‐up PCR product.

166 Submit ∼500 ng sample for Illumina deep sequencing.
  Final concentration should be ∼5 to 10 ng/µl.
  Ideally sequence paired‐end. Single‐read sequencing is also possible at the expense of the loss of fragment‐length information (for analysis, reads should be extended by the median size of the STARR‐seq plasmid library; see Basic Protocol 1, step 12). Use the same sequencing mode for STARR‐seq screen and STARR‐seq plasmid library (input; Basic Protocol 1, step 126).
167 Store sample at −20°C.

## UMI‐STARR‐seq SCREENING PROTOCOL: UNIQUE MOLECULAR IDENTIFIER INTEGRATION

This protocol is an addition to the standard STARR‐seq protocol (Basic Protocol 2) and explains how to introduce unique molecular identifiers (UMIs) into the reporter transcripts prior to amplification (Fig. 1, right). This allows the counting of individual reporter transcripts, which is preferable when performing STARR‐seq with low‐complexity candidate libraries that are prone to PCR‐amplification biases, especially when individual candidates or defined synthetic oligos are screened. Note that a spike‐in control might be needed for normalization (see Basic Protocol 2, steps 47 to 62, spike‐in control for library normalization.

As this protocol is a variant of Basic Protocol 2, only steps that deviate are described (Fig. 1, right). Please follow Basic Protocol 2 until RNaseA treatment (step 112).

During this protocol, indexing of different STARR‐seq screens is only possible using the i5 index, as the i7 index is replaced with the UMI, making it possible to count individual reporter transcripts. It is important to note that the UMI will be read during deep sequencing on an Illumina platform as Index 1 (i7) read. This requires that the Index 1 (i7) read be read for 11 cycles (instead of the standard 8 cycles) to account for the UMI length (10 nucleotides).

For the sequencing‐ready PCR, replace the Illumina i7 index primer with P7‐seqReady rev primer (10 µM) (CAAGCAGAAGACGGCATACGAGA*T).

### Additional Materials (also see Basic Protocol 2)

Primers:
  Second strand primer: GTCGTGAGGCACTGGGCA*G
  P7‐UMI‐primer: CAAGCAGAAGACGGCATACGAGAT**NNNNNNNNNN**GTGACTGGAGTTCAGACGTGT*G
  P7‐junction rev primer: CAAGCAGAAGACGGCATACG*A
  P7‐seqReady rev: CAAGCAGAAGACGGCATACGAGA*T
  * = phosphorothioate bond (protection of primer from 3′ to 5′ exonuclease activity of proof‐reading DNA polymerase; especially important for junction fw primer that specifically binds across the splice junction of the reporter transcripts/cDNA)

*NOTE*: This protocol is identical to Basic Protocol 2 until step 112, and only deviates from “cDNA purification after RNaseA treatment” on. Please follow Basic Protocol 2, steps 1 to 112, and then continue with this Alternate Protocol starting from step 1. After step 16, switch back to Basic Protocol 2 and continue from step 131, with the exception of using the UMI‐STARR‐seq‐specific primers (see Alternate Protocol).

### cDNA purification after RNaseA treatment

Before starting:

Prepare 80% ethanol (diluted with nuclease‐free MonoQ H_2_O)
Warm AMPureXP beads to room temperature
Resuspend AMPureXP beads thoroughly by vortexing before use.

1 Pool all cDNA samples, mix thoroughly, and distribute 200 µl (=10 RT reactions; keep minus RT separate) to 1.5‐ml LoBind tubes.
2 Add 1.4 vol beads to 1 vol of cDNA (add 280 µl beads to 200 µl cDNA; 28 µl to minus RT) in 1.5‐ml LoBind tube, briefly vortex, then pipette up and down 20 times to mix.
3 Incubate 15 min at room temperature.
4 Transfer to magnetic separator and incubate 10 min at room temperature.
  Keep tubes/samples on magnetic separator for the following steps (except elution); pipette onto opposite tube wall.
5 Remove all liquid.
6 Wash twice with 1 ml 80% ethanol, incubating 2 min (while on magnet) each time.
  Make sure that beads are covered completely.
7 After second wash, make sure to remove 80% ethanol completely.
8 Dry beads at room temperature to allow evaporation of remaining 80% ethanol for 5 to 10 min.
  Keep tubes on magnet with open lids. Avoid over‐drying (elute as soon as the bead pellet starts to show cracks).
9 To elute, add 43 µl nuclease‐free H_2_O per five RT reactions (86 µl per tube), close tube, and remove from magnet.
10 Resuspend beads completely by vortexing thoroughly and incubate at 37°C on thermomixer for 3 min while shaking (300 rpm).
11 Transfer beads/tubes immediately onto magnetic separator and carefully tilt magnet to allow the beads to migrate upwards on wall. Incubate for 1 min.
12 Transfer supernatant/sample to new tube.
13 Pool all cleaned‐up cDNA samples (except minus RT).

### Second strand synthesis

During this reaction, the second DNA strand of the reporter cDNA is synthesized by a linear PCR reaction without amplification, using a DNA polymerase (KAPA HiFi HotStart DNA Polymerase) and a reporter transcript–specific forward primer. The resulting double‐stranded reporter DNA will then be competent for UMI introduction.

14 Prepare the master mix for the second‐strand synthesis.
  Process the entire pool of cDNA. Scale the master mix accordingly.
  Second‐strand synthesis mix (for one reaction):
  42.5 µl cDNA (=5 RT reactions)
  50 µl KAPA 2 × HiFi Hot StartReady Mix
  7.5 µl second‐strand primer (10 µM) (GTCGTGAGGCACTGGGCA*G)
  Perform the following thermal cycling program:

**Table:** 

Denaturation:	98°C for 60 s
Annealing:	65°C for 30 s
Elongation:	72°C for 90 s.
  The elongation time depends on the library insert size.

### cDNA purification

Please follow Alternate Protocol, steps 1 to 13.

### Unique molecular identifier introduction (UMI‐PCR)

This reaction adds a UMI to each reporter transcript by a linear PCR reaction (Fig. 1, right). To introduce the UMI, we use a modified Illumina i7 index primer containing 10 random nucleotides at the position of the Illumina i7 index. The UMI‐containing primer binds to the reverse Illumina adapter (P7) and replaces the original Illumina i7 index (Illumina i7 primer) that binds at this position in Basic Protocol 2 step 150. This makes it possible to read the UMI during Illumina sequencing as Index 1. As the UMI is 10 nucleotides long, the Index 1 during Illumina sequencing needs to be read for 11 bp.

Note: the UMI introduction is also possible at the RT step but is not recommended as it leads to reduced specificity towards the STARR‐seq reporter transcripts.

15 Prepare the master mix for the UMI ‐PCR (linear UMI‐PCR reaction mix):
  42.5 µl cDNA (second‐strand cDNA =five RT reactions)
  50 µl KAPA 2× HiFi Hot Start ready Mix
  7.5 µl P7‐UMI primer (10 µM) **(**CAAGCAGAAGACGGCATACGAGAT**NNNNNNNNNN**GTGACTGGAGTTCAGACGTGT*G**)**
  Perform the following thermal cycling program:

**Table:** 

Denaturation:	98°C for 60 s
Annealing:	65°C for 30 s
Elongation:	72°C for 90 s.

### cDNA purification (UMI‐PCR)

Please follow Alternate Protocol, steps 1 to 13.

16 Elute in 20 µl nuclease‐free H_2_O per RT reaction (100 µl per tube).

### Junction PCR

See Basic Protocol 2, step 127, for the junction PCR master mix; use 20 l UMI‐PCR from step 16 as template. Replace the junction rev primer with the P7‐junction rev primer (10 µM) (CAAGCAGAAGACGGCATACG*A) using the following PCR program:

**Table:** 

1 cycle:	45 s	98°C	(denaturation)
16 cycles:	15 s	98°C	(denaturation)
	30 s	65°C	(annealing)
	70 s	72°C	(extension)
1 cycle	2 min	72°C	(final extension).

### Purification of jPCR

Please follow Basic Protocol 2, steps 131 to 143.

### Sequencing‐ready PCR

Please follow Basic Protocol 2 from here on—“Sequencing ready PCR”—step 144.

Indexing of STARR‐seq screens can only be done using the i5 index as the i7 index is replaced by the UMI.

For the sequencing‐ready PCR, replace the Illumina i7 index primer with P7‐seqReady rev primer (10 µM) (CAAGCAGAAGACGGCATACGAGA*T).

## TRANSFECTION OF HUMAN CELLS USING THE MaxCyte STX SCALABLE TRANSFECTION SYSTEM

This protocol describes the transfection of human cells by electroporation using the MaxCyte STX scalable transfection system. This is the standard protocol we use in our lab for the electroporation of STARR‐seq plasmid libraries into human and Drosophila cells. This support protocol yields very high transfection efficiency (>60% to 90%) and cell viability (75% to 90%).

### Additional Materials (also see Basic Protocol 2)

Electroporation buffer (MaxCyte cat. no. EPB1),
Clinical Processing Assembly, OC‐100 100‐µl (MaxCyte cat. no. GOC1)
Clinical Processing Assembly, OC‐400 400‐µl (MaxCyte cat. no. GOC4)

1 Grow cells and split 24 hr before transfection to reach 4 × 10^8^ cells at 80% confluency on electroporation day.
  For genome‐wide screens, use 4 × 10^8^ per replicate; for focused screens (BAC screens), use 8 × 10^7^ cells. These numbers were determined in HCT116 cells (transfection efficiency >80%, viability >90%). The number of cells may vary, depending on cell line and transfection method.
2 Remove the medium completely.
3 Wash cells with 1× PBS then remove PBS completely.
  For adherent cells: add trypsin and incubate at 37°C for 4 to 5 min. Add growth medium and resuspend cells by pipetting up and down thoroughly.
4 Collect the cells from all plates/flask and count cells using automated cell counter.
5 Transfer 4 × 10^8^ cells (for genome‐wide screen) to a 50‐ml tube (calculate corresponding volume according to cell counts). For focused screen, use 8 × 10^7^ cells.
6 Spin down cells for 5 min at 125 × *g*.
7 Remove medium, resuspend cell pellets in ∼10 ml electroporation buffer, and pool all cells.
8 Spin down cells 5 min at 125 × *g*.
9 Remove electroporation buffer and add the STARR‐seq plasmid library to the loosened cell pellet [library concentration needs to be >1.0 µg/µl in H_2_O (H_2_O is mandatory)].
  Use 80 µg library per 4 × 10^7^ cells.
  For a genome‐wide screen, use a total of 800 µg STARR‐seq plasmid library and 4 × 10^8^ cells.
10 Fill up the cell/library mix with electroporation buffer (account for the previous added STARR‐seq library and cell pellet) to a final volume of 4 ml.
11 Use cell‐line specific pre‐set transfection protocol of the MaxCyte STX scalable transfection system. Electroporate in steps of 400 µl (for genome‐wide screen, 10 steps, and for focused screen, 2 in total) using OC‐400 processing assemblies (cuvettes). Transfer all cells after electroporation into a single pre‐warmed T‐225 flask.
12 Allow cells to recover for 25 min post‐electroporation without adding growth medium at 37°C.
  The recovery of the electroporated cells without growth medium is critical for optimal viability and transfection efficiency.
13 Transfer the T‐225 flask containing the electroporated cells (without medium) to a 37°C incubator for 25 min.
14 After 25 min, add 350 ml growth medium without antibiotics to the recovered cells (focused screen 70 ml).
  For cells with interferon (INF) response, add 350 µl (70 µl for focused screen) of a 1 mM stock of C16 and BX‐795 inhibitors to the cells (final concentration 1 µM/inhibitor).
15 Plate the transfected cells onto five square plates, with 70 ml/square plate (24.5 × 24.5 cm) corresponding to 80 million cells/square plate. For a focused screen, plate the cells on one square plate.
16 Incubate cells for 6 hr, i.e., lyse cells 6 hr after addition of the growth medium (step 14).
  To ensure that the cells can be lysed after 6 hr, we recommend that harvesting of the cells be started already after 5 hr when performing genome‐wide screens.
17 Continue with Basic Protocol 2 step 20—harvesting of cells.

## REAGENTS AND SOLUTIONS

### Diethylpyrocarbonate (DEPC) treatment of solutions

Add 0.2 ml DEPC to 100 ml of the solution to be treated. Shake vigorously to get the DEPC into solution. Autoclave the solution to inactivate the remaining DEPC. Many investigators keep the solutions they use for RNA work separate to ensure that “dirty” pipets do not go into them.

CAUTION: Wear gloves and use a fume hood when using DEPC, as it is a suspected carcinogen.

### Regeneration of Dynabeads Oligo‐dT_25_ for re‐use

Reconditioning and storage buffers (see recipes) need to be at room temperature before use.

1. Resuspend beads by vortexing thoroughly.
2. Transfer Dynabeads Oligo‐dT_25_ onto magnetic separator and incubate for 1.5 min. Make sure that solution is clear.
3. Remove supernatant completely.
4. Add one volume of reconditioning buffer (see recipe) to the beads. The volume refers to the starting volume of beads (see Basic Protocol 2, steps 63 to 82).
5. Incubate at 65°C for 10 min on heating block while shaking at 300 rpm.
6. Transfer beads to magnetic separator, incubate for 1 min, and discard the supernatant.
7. Wash twice with 1 vol reconditioning buffer (see recipe). Incubate on magnet for 1.5 min.
8. Wash 3×, each time with 1 vol storage buffer. Incubate on magnet for 1.5 min.
9. Resuspend Dynabeads Oligo‐dT_25_ in 1 vol storage buffer and transfer to a new tube.
10. Store Dynabeads Oligo‐dT_25_ at 4°C. Dynabeads Oligo‐dT_25_ can be re‐used according to manufacturer's instructions.

### Buffers for Dynabeads Oligo‐dT_25_

See Table 1.

**Table 1:** Buffer Preparation

	Stock	Binding buffer (2.5 × per screen)		Washing buffer (2 × per screen)		Storage buffer (5 × per screen)		Reconditioning buffer (3 × per screen)		10 mM Tris·Cl	
Tris·Cl, pH 7.5	1 M	20 mM	10 ml	10 mM	5 ml	250 mM	125 ml			10 mM	1 ml
LiCl	5 M	1 M	100 ml	0.15 M	15 ml						
EDTA, pH 8	0.5 M	2 mM	2 ml	1 mM	1 ml	20 mM	20 ml				
Tween 20						0.10%	500 μl				
NaN_3_	10%					0.02%	1 ml				
DEPC‐treated MonoQ H_2_O*a*			388 ml		479 ml		353.5 ml		490 ml		99 ml
NaOH	5 M							0.1 M	10 ml		
			500 ml		500 ml		500 ml		500 ml		100 ml

^*a*^DEPC‐treated MonoQ H_2_O: add 750 µl DEPC to 500 ml MonoQ H_2_O and incubate overnight. Autoclave to inactivate DEPC.

## COMMENTARY

### Background Information

Genome‐wide association studies (GWAS) suggest that >80% of disease‐associated variants lie within non‐coding regions of the genome that likely harbor enhancers (Corradin & Scacheri, 2014). Therefore, the identification of enhancers and the assessment of their activities is of critical interest. As enhancers retain their activity outside their endogenous context, plasmid‐based reporter assays are widely used as the gold standard to validate single enhancers and confirm their enhancer activity [but see Catarino and Stark (2018) for a discussion of ectopic versus endogenous enhancer activities]. In order to test multiple enhancer candidates simultaneously, massively parallel reporter assays (MPRAs) have been developed that use different strategies, often employing unique DNA sequences as molecular barcodes [see Inoue and Ahituv (2015) and Santiago‐Algarra et al., (2017) for reviews]. In STARR‐seq (Arnold et al., 2013), the enhancer candidate fragments themselves are cloned downstream of a core promoter (fly STARR‐seq) or the bacterial ORI, which serves as core promoter in human STARR‐seq. This arrangement means that active enhancers transcribe themselves as part of the reporter transcript and serve as their own barcodes, enabling the large‐scale identification of enhancers on a genome‐wide scale and the quantification of their strength by deep sequencing.

STARR‐seq was originally developed in *Drosophila* S2 cells and adapted to focused libraries in human cells in 2013 (Arnold et al., 2013), and more recently revised to enable genome‐wide screening in human cells. The latter was made possible by resolving two issues that impact all plasmid‐based transcriptional reporter systems in human cells (Muerdter et al., 2018): (1) in mammalian cells (but not in fly cells), the bacterial “origin of replication” (ORI) functions as a core promoter and initiates reporter transcription (Lemp et al., 2012); and (2) many human cell lines induce a strong interferon response against dsRNA and cytoplasmic DNA, which occurs during reporter plasmid transfection. To avoid this scenario, key kinases involved in interferon induction can be specifically inhibited (Chen et al., 2016; Nejepinska et al., 2014; Paludan & Bowie, 2013).

### Critical Parameters

#### Efficiency of STARR‐seq plasmid library cloning

To ensure the highest efficiency during library cloning, closely follow Basic Protocol 1, including all recommendations. All cleanup steps throughout Basic Protocol 1 are essential for maximum library complexity. For example, if not column purified, the PCR‐amplified library insert (after AMPureXP size selection) as well as the In‐Fusion HD cloning reaction negatively impact the cloning efficiency. The most critical step during library cloning is the transformation of the library cloning reaction into bacteria. We highly recommend using MegaX DH10B™ T1R electrocompetent bacteria (Invitrogen cat. no. C640003) and following all steps in Basic Protocol 1 to achieve maximum efficiency and complexity of the STARR‐seq plasmid library.

#### Transfection efficiency of human cells

The transfection efficiency and cell viability should be as high as possible, to ensure high coverage and complexity of reporter transcripts. Optimize the transfection protocol for each cell line prior to performing STARR‐seq. Low transfection efficiency could limit the performance of STARR‐seq.

#### Type‐I interferon response upon plasmid transfection

Plasmid transfection (including STARR‐seq library transfection) triggers a type‐I interferon response in many mammalian cell lines, which substantially impacts gene regulation and can lead to confounding false‐positive and false‐negative STARR‐seq signals. This can be prevented using inhibitors for key signaling kinases (PKR and TBK1; only applicable for cell lines that activate an interferon response).

#### Harvesting of adherent cells post transfection

If adherent cells are harvested 6 hr post electroporation of the STARR‐seq plasmid library, a considerable fraction is typically not yet attached to the culture flask. Therefore, care should be taken not to discard cells in the culture medium and washing solution, to ensure maximum recovery of STARR‐seq library–transfected cells.

#### Degradation of RNA

Ensure RNase‐free working environment and reagents throughout Basic Protocol 2 and the Alternate Protocol to prevent RNA degradation, which would result in the loss of STARR‐seq reporter mRNAs.

#### PCR amplification biases

To prevent PCR amplification biases, it is critical to use the KAPA HiFi DNA Polymerase (KAPA 2× HiFi HotStart Ready Mix; KAPA Biosystems cat. no. KK2601) for all PCR amplification steps throughout Basic Protocols 1 and 2 as well as the Alternate Protocol. PCR amplification biases would otherwise negatively impact library cloning and STARR‐seq.

#### Discrimination between STARR‐seq plasmid and reporter transcript/cDNA

To discriminate between STARR‐seq library plasmids and reporter transcript cDNAs (Basic Protocol 2, steps 127 to 130), we included an intron in the STARR‐seq screening plasmid. We ensure that only spliced reporter cDNAs are amplified during junction PCR by using a primer that exclusively binds to the spliced reporter cDNA across the exon–exon splice junction but not the intron‐containing plasmid DNA. This makes it possible to specifically enrich reporter cDNAs but not residual library plasmids.

Note that the junction PCR is indispensable, as STARR‐seq plasmids can still be detected in the RNA sample even after Turbo DNase treatment (for junction PCR, see Basic Protocol 2, steps 127 to 130).

#### Number of PCR cycles for final amplification (sequencing‐ready PCR)

After the selective amplification of reporter cDNA in a first PCR step (junction PCR, see Basic Protocol 2, steps 127 to 130), the candidates are further amplified for deep sequencing (sequencing‐ready PCR). The extent of amplification during this second step is critical, and the optimal number of PCR cycles needs to be experimentally determined for every STARR‐seq screen (test PCR, Basic Protocol 2, steps 144 to 149). It is important to avoid over‐amplification while at the same time obtaining enough material for deep sequencing (200 to 1000 ng)—we achieve this typically with five to eight PCR cycles. An unexpected high number of PCR cycles (>12) determined during the test PCR may indicate low complexity of the STARR‐seq cDNA pool and failure of STARR‐seq. The major cause for this scenario is poor transfection performance.

### Troubleshooting

See Table 2 for troubleshooting suggestions.

**Table 2:** Troubleshooting

Problem	Possible cause	Solution
Low number of cells	Cells have too low density on transfection day	Check growth rate of cell line. Seed more densely prior to transfection.
Low complexity of STARR‐seq plasmid library	Inefficient In‐Fusion HD reaction or reaction cleanup Low efficiency of bacterial transformation	Follow “In‐Fusion HD reaction” steps of Basic Protocol 1 closely Use recommended electrocompetent bacteria Determine and ensure maximum efficiency of bacteria transformation Check complexity of cloned library before use by deep sequencing Try Gibson Assembly for library cloning (some inserts work better with Gibson cloning)
Low transfection efficiency of human/mammalian cells	Wrong transfection conditions Low viability of cells Issue with culturing of cells Issue with transfection device/method	Optimize transfection conditions (manufacturer's guidelines!) Optimize culturing of cells
High number of PCR cycles for “sequencing‐ready PCR” (>12 cycles)	Low transfection efficiency Inefficient cell lysis Degradation of RNA (incl. reporter transcripts) Loss of sample during protocol	Monitor transfection efficiency by FACS (GFP control transfection) Homogenize cells for 4.5 min Ensure RNase‐free working environment
Low complexity of STARR‐seq screen (after deep sequencing)	Low transfection efficiency Inefficient cell lysis Degradation of RNA (incl. reporter transcripts) Loss of sample during protocol Sequencing depth low	Ensure sufficient number of reads; Confirm input library complexity by deep‐sequencing Use UMI‐STARR‐seq protocol (Alternate Protocol) for low‐complexity STARR‐seq plasmid libraries

### Statistical Analyses

Data processing and statistical analyses are very similar to other deep‐sequencing based approaches that enrich for specific sequences (peaks) compared to overall genomic coverage, i.e., ChIP‐seq, ATAC‐seq, DNase‐seq, MNase‐seq.

### Data Visualization

Map single or paired‐end reads to the respective reference genome (recommended tool: “Bowtie 1”) and create position‐specific read‐coverage tracks (bigwig format), which can be visualized using the UCSC genome browser in the context of annotated genes and published datasets (e.g., from ENCODE). Visually inspect the respective tracks (see Understanding Results for details). Confirm successful performance of STARR‐seq by the presence of positive control peaks (if included in library).

To call STARR‐seq peaks over input, use MACS2 or any other peak‐calling algorithm that is commonly used for ChIP‐seq or ATAC‐seq. The STARR‐seq sample corresponds to the reporter‐transcript‐derived sequencing reads as obtained via Basic Protocol 2, and the input is generated by deep sequencing of the STARR‐seq plasmid library. For this, the STARR‐seq plasmid library is amplified using the Illumina i5 and i7 index primers before transfection (Basic Protocol 1, steps 112 to 126). We found no difference between sequencing the transfected DNA and original library and conclude that is not necessary to extract plasmid DNA from transfected cells for normalization (Arnold et al., 2013). The peak height (STARR‐seq signal at peak summits over input) corresponds to enhancer activity.

### Understanding Results

Evaluate the read coverage profile for the plasmid library (input). The read coverage profile should appear smooth if coverage is sufficiently high.

Peaks (enhancers) should have a bell shape rather than a rectangular appearance—the latter indicates sparse stochastic signals that could stem from a low‐complexity input library or poor STARR‐seq performance dominated by PCR‐amplification artifacts. This becomes especially important if using all reads for analysis—instead of position‐collapsed reads—to discriminate between peaks that stem from enhancer activity and peaks created by PCR duplications. Only “peaks” from low‐complexity libraries cloned from individual enhancers or synthesized DNA oligo pools should appear rectangular, as every position is usually covered by only one fragment. Quantification of such low‐complexity libraries should be done using UMI‐STARR‐seq.

### Time Considerations

#### STARR‐seq screening protocol

Seed cells 24 to 48 hr before transfection to reach 80% confluency on the day of transfection. For focused (BAC) screens, use 8 × 10^7^ cells; for a genome‐wide screen use at least 4 × 10^8^ cells. The time from starting the transfection of the STARR‐seq library into cells until isolation of total RNA from the transfected cells is 6 hr.

Recommended: Start with transfection of cells in the morning and start harvesting cells after 5 hr (for a genome‐wide screen, harvesting cells takes ∼1 hr) to lyse cells after 6 hr. After total RNA isolation, the samples can be frozen at −80°C. The STARR‐seq screening protocol (Basic Protocol 2) can be conducted in 1½ days [if using the UMI‐STARR‐seq protocol (Alternate Protocol), 2 days]. We recommend processing mRNA to cDNA on one single day to reduce the risk of RNA degradation. cDNA processing and library amplification takes around 1/2 day.

#### STARR‐seq library cloning protocol

The STARR‐seq library cloning protocol (Basic Protocol 1) takes approximately 1½ days. On day 1, the STARR‐seq reporter plasmid is digested and cleaned up, the candidate library insert is generated and amplified, and the library cloning reaction is performed, cleaned up, and transformed into highly competent bacteria. The electroporated bacteria are inoculated into liquid cultures and grown overnight. On the second day the bacteria are harvested, and the STARR‐seq plasmid library is prepared from bacterial pellets.
